# A novel conductive microtubule hydrogel for electrical stimulation of chronic wounds based on biological electrical wires

**DOI:** 10.1186/s12951-024-02524-2

**Published:** 2024-05-16

**Authors:** Weijing Fan, Xiao Yang, Xiaoming Hu, Renyan Huang, Hongshuo Shi, Guobin Liu

**Affiliations:** Department of Vascular Surgery, Shuguang Hospital Affiliated to Shanghai University of Traditional Chinese Medicine, Zhangheng Street, Pu Dong New District, Shanghai, 201203 China

**Keywords:** Chronic wounds, Electrical stimulation, Conductive dressing, Microtubules, Biological electrical wires, MT-MAA hydrogel

## Abstract

Electrical stimulation (ES) is considered a promising therapy for chronic wounds via conductive dressing. However, the lack of a clinically suitable conductive dressing is a serious challenge. In this study, a suitable conductive biomaterial with favorable biocompatibility and conductivity was screened by means of an inherent structure derived from the body based on electrical conduction in vivo. Ions condensed around the surface of the microtubules (MTs) derived from the cell’s cytoskeleton are allowed to flow in the presence of potential differences, effectively forming a network of biological electrical wires, which is essential to the bioelectrical communication of cells. We hypothesized that MT dressing could improve chronic wound healing via the conductivity of MTs applied by ES. We first developed an MT-MAA hydrogel by a double cross-linking method using UV and calcium chloride to improve chronic wound healing by ES. In vitro studies showed good conductivity, mechanical properties, biocompatibility, and biodegradability of the MT-MAA hydrogel, as well as an elevated secretion of growth factors with enhanced cell proliferation and migration ability in response to ES. The in vivo experimental results from a full-thickness diabetic wound model revealed rapid wound closure within 7d in C57BL/6J mice, and the wound bed dressed by the MT-MAA hydrogel was shown to have promoted re-epithelization, enhanced angiogenesis, accelerated nerve growth, limited inflammation phases, and improved antibacterial effect under the ES treatment. These preclinical findings suggest that the MT-MAA hydrogel may be an ideal conductive dressing for chronic wound healing. Furthermore, biomaterials based on MTs may be also promising for treating other diseases.

**Supplementary Information:**

The online version contains supplementary material available at 10.1186/s12951-024-02524-2.

## Introduction

Chronic wounds are a common health burden, especially for diabetic and paralyzed patients. Literatures have described that chronic wounds have the same impact on patients’ quality of life as kidney and cardiovascular diseases. For some patients suffering from chronic wounds, the current mortality rate is comparable to that of cancer patients. The global market of advanced wound care products is growing rapidly, about $12 billion in 2020 and estimated to reach $18.7 billion by 2027 [1].

The physiological process of wound healing includes four steps: Hemostasis, inflammation, proliferation and maturation, which involve the coordinated activities of various cells, proteins, and signaling molecules, and only when these steps proceed in a correct and well-coordinated manner, can proper healing be realized. However, the failure to proceed through this organized process will delay the healing of skin tissue, eventually resulting in chronic wounds. It may take more than three months for chronic wounds to heal, which are typically accompanied by inflammation and noticeable scarring [2]. Various strategies have been developed for chronic wound healing, such as the application of growth factors [3], ultrasound [4], tissue engineering [5], wound dressings [6] and stem cells [7]. Among these methods, the use of autologous skin grafts is considered the preferred method as well as *a* gold standard. However, this method has some limitations as it is not suitable for wounds that exceed 60% of the patient’s total body surface area. Inadequate and delayed dermal regeneration at the tissue harvesting site may lead to severe scarring. The approaches such as nanotechnology, tissue engineering and stem cells, etc. suffer from the shortcomings of infection risks, slow healing rates and poor biocompatibilty [8]. At present, chronic wound healing remains to be a challenging issue in both clinic and scientific research.

Conductivity is an important characteristic of skin, hence the name “skin battery”, which is essential to the function of skin [9]. Endogenous electric fields (EFs) are generated by skin injury, and they can promote wound healing [10]. Since the 21st century, exogenous electrical stimulation (ES) has been studied and applied for chronic wound healing, improving all stages of wound healing by various pathways and mimicking the EF of wounds [11]. It can alleviate edema around the electrode, guide keratinocyte migration, enhance re-epithelialization, direct dermal angiogenesis, modulate a variety of genes relevant with wound healing, and generate antibacterial effects. For example, Josef M. Penninger et al. reported that ES can provide directional cues for cell movement during wound healing through phosphatidylinositol-3-OH kinase-γ and PTEN. Some international clinical guidelines published by bodies such as the Australian Wound Management Association and the Consortium for Spinal Cord Medicine suggest the use of ES to help promote chronic wound healing. However, the therapeutic effect of ES on chronic wound healing is restricted by the spread of ES, which results from significant exudates, uneven shapes, and decreased conductivity of the wound bed [12]. To date, conductive wound dressings have been developed and used to enhance the therapeutic effect of ES, attracting much attention in chronic wound healing [13].

Favorable conductive wound dressings mimicking the conductivity of skin show promising potential in accelerating chronic wound healing and skin tissue regeneration when applied by ES [14, 15]. The common conductive materials used for wound dressing mainly include carbon-based materials (such as PPy/CNT/PU and CNTs) [13], polymer biomaterials (such as PANI-GCS and MXene/cellulose) [16], and metal materials (such as Ag, zinc oxide, and Ti3C2Tx) [17]. For example, Ali Khademhosseini et al. developed a flexible patch with silver nanowires for wound healing by ES [18]. Peter X Ma and his colleagues reported that conductive hydrogels based on carbon nanotubes could promote wound healing [19]. However, there is currently no ES dressing approved by the FDA to accelerate wound healing. It has therefore become a hot research topic to screen a favorable conductive biomaterial for chronic wound healing [15].

In this study, a clinically suitable conductive biomaterial with good conductivity, biocompatibility and biodegradability was screened by means of an inherent structure derived from biomolecular structures based on electrical conduction in vivo. Known as “biological electrical wires”, MTs can transmit and amplify electric signals via the condensed ionic current along MTs [20], which is essential to the bioelectrical communication of cells [20, 21]. Additionally, MTs are part of the cell’s cytoskeleton with good biocompatibility and biodegradability.

MTs have a hollow tube structure composed of 13 protofilaments polymerized by the α-tubulin and β-tubulin proteins. MTs possess the electrical characteristics of biological semiconductors and memristors due to their unique structures [21]. Direct measurements of conductivity along MTs in solution were 90 S/m using microchannels (150 mS/m using the electroorientation method) [22]. Ionic conductivity assays reveal that the MT solution has a higher conductivity than a simple solution, and the direct current is enhanced by as much as three times in the MT solution when a pulse voltage is applied across a 25–60 μm distance. The conductivity of MTs is increased by 15× compared to that of the surrounding solution [23]. MTs at alternating current show a higher conductivity than those at direct current. The water channels inside the MTs may be mainly responsible for the high conductivity at alternating current frequencies [23]. It is also worth noting that the conductivity of MTs is sensitive to environmental factors, e.g., temperature, pH, and ionic species [22, 24]. MTs can be a responsive conductive biomaterial in different ambient environments.

It is therefore essential to find a suitable fabrication approach for MTs-loaded wound dressings that are characterized by good structural morphology, biocompatibility and biodegradability for chronic wound healing [25]. Wound dressings can be prepared by conductive materials in various forms for different wounds. Two-dimensional dressings are used for simple wounds with less exudates (such as film and fibers), and three-dimensional dressings can be applied to chronic wounds and severe skin defects (such as hydrogel and foam) [15, 26, 27]. Hydrogels are suitable for wounds with excessive exudates due to their capacity for water absorption. Moreover, hydrogels can be loaded with bioactive molecules and therapeutic cells, showing great advantages in healing complicated skin injuries [28]. As a natural biomaterial composed of mannuronic acid and guluronic acid [29], alginate and its derivatives have been widely applied in biomedical fields for decades due to their good biocompatibility, specific bioactivity, and exudate absorption ability [30–32]. The list of commercially available alginate and its derivative-based wound dressings is growing rapidly [29, 32]. Methacrylated alginate (MAA) is a common derivative of alginate. MAA hydrogels have been studied and clinically used in wound dressings, with structural features similar to those of the native extracellular matrix (ECM) [26, 27], and this is a highly desirable property for treating chronic wounds [33].

In this study, we first developed a novel conductive hydrogel using MAA and MTs. The MT-MAA hydrogel was prepared by a double cross-linking method in vitro. The purpose of this study was to determine whether the MT-MAA hydrogel is a good conductive dressing applied by ES for chronic wound healing. MTs act as bioelectrical wires for the electrical communication of cells. When the MT-MAA hydrogel was implanted into chronic wounds, ES enhanced wound healing by improving the conductivity of MTs (Graphical Abstracts). The results suggest that the MT-MAA hydrogel may be a promising conductive dressing for treating chronic wounds.

## Materials and methods

### Preparation of the MT-MAA hydrogel using MTs and MAA

#### Preparation of MTs

Tubulin and MTP were purchased from cytoskeleton Inc. (Denver, CO, USA), and Taxol was purchased from Sigma‒Aldrich (Shanghai, China). Microtubules (MTs) were prepared as described in previous reports with modifications [34]. Briefly, tubulin was dissolved in 65 mM PIPES (pH 7.0), 1 mM MgCl_2_, 1 mM EGTA, 1 mM GTP, and 5% glycerol (Sangon, Shanghai, China). MTs were polymerized by tubulin solution (6 mg/ml) at 36 °C for 28 min, and the polymerization of MTs was maintained by adding 20 mM Taxol. Excess Taxol was removed by sedimentation at 30,000 × g for 35 min. MTs were suspended in PBS buffer free from Taxol. Depolymerization and polymerization of MTs were assayed using the CytoDYNAMIX Screen kit at 350 nm by a plate reader (Fluoroskan FL, Thermo Scientific™. USA, repeated three times).

#### Preparation of the MT-MAA hydrogel and MAA hydrogel with different formulas

The hydrogel was prepared as described in a previous study [18]. Briefly, methacrylic acid alginate (MAA, 50 kDa, purchased from Sigma‒Aldrich, Shanghai, China) was dissolved in NaOH (0.1 M, Sigma‒Aldrich, Shanghai, China), dialyzed (MWCO 10 kDa) with distilled water overnight, and lyophilized. MAA hydrogels of different concentrations were prepared (0.5, 1, 1.5, 2 MAA%). We developed MT-MAA hydrogels with different formulas (0.5, 0.75, 1, 1.25, 1.5, 1.75, 2 MT%):2 (MAA%). The MT-MAA hydrogel was fabricated by a double cross-linking method using UV (5.0 mW cm^− 2^, 3 min. UV-crosslinker, Spectronics, USA) and calcium chloride (20 mmol/L, 10 min. Sigma‒Aldrich, Shanghai, China). Then, the hydrogel was soaked in excessive PBS to remove calcium chloride.

### Characterization of the MT-MAA hydrogel

#### Morphology and structural characteristics of the MT-MAA hydrogel and MTs

Scanning electron microscopy (SEM), transmission electron microscopy (TEM) and fluorescence microscopy were adopted to show the morphology and structure of MTs. SEM was used to show the structure of the MT-MAA hydrogel. The protocols used for these studies are presented in the supporting information.

#### Conductivity experiment of the MT-MAA hydrogel and MTs

As previously reported, a two-probe method was used to measure the conductivity of the MT-MAA hydrogel (each *n* = 8) [35]. Briefly, the samples (L = 2 mm, D = 10 mm) were immersed in PBS solution (pH = 7.4) for 18 h, and then two parallel titanium electrodes were fixed onto the ends of the samples to test the conductivity (FE38-Standard, METTLER TOLEDO, Shanghai, China). The conductivity of the samples was calculated by Equation ${\sigma }=\frac{4\text{I}\text{l}}{\text{V}\text{*}{\Pi }\text{d}{\text{x}}^{2}}$(σ, S/cm). The dressings were excised in vivo from the wound area within 14 d (each *n* = 8), and the conductivity was measured as described above (repeated three times).

Under various conditions (pH, ionic species), the conductivity of MTs was measured by the electroorientation method as previously reported [24]. The protocols are presented in the supporting information.

#### Mechanical property test of the MT-MAA hydrogel

The compression and tension properties (mechanical properties) of the samples were tested using a universal testing machine (UTM-1004E 50 KN, Gotester Machines Co., Ltd, USA). The samples were prepared in a cube shape (length: 1 cm, width: 1 cm and thickness: 1 cm) for the compression and tension tests. The compression and tension of samples were conducted to the maximum strain (speed: 2 mm/min, each *n* = 6). The modulus of the mechanical property was measured from the initial linear segment corresponding to 0–25% strain. Every sample was tested three times.

#### Cell culture and biocompatibility of the MT-MAA hydrogel

Mesenchymal Stem Cells (MSCs) were derived from sacrificed C57BL/6J mice (6–8 weeks) and cultured with MSC-GM medium (Cyagen, Guangzhou, China) using the whole bone marrow adherence method at 37 °C in humidified 5% CO_2_. Undifferentiated MSCs derived from the third passage were used in the experiments for this study. Human umbilical vein endothelial cells (ECs) and NIH3T3 fibroblast cells (NIH3T3 cells) were purchased (Cyagen, Guangzhou, China) and cultured in 1640 medium containing 10% fetal calf serum (HyClone, USA) at 37 °C in a humidified atmosphere with 5% CO_2_.

Biocompatibility was studied by apoptosis of NIH3T3 cells using flow cytometry. MT-MAA hydrogel (500 µl) was sprayed onto 6-well plates (Cyagen, Guangzhou, China) and incubated at 37 °C for 20 min. NIH3T3 cells (1 × 10^5^/ml, 1.5 ml) were seeded and cultured in 6-well plates, where the cell culture media (1640 medium containing 10% fetal calf serum) was refreshed every day. NIH3T3 cells were harvested, resuspended in PBS, and washed three times with PBS at 1 d, 3 d, 5 d, and 7 d (each *n* = 8). Using the Annexin V-FITC/PI Apoptosis Detection Kit (Sigma‒Aldrich, CA, USA) according to the instructions, NIH3T3 cells were analyzed by flow cytometry (FACSVerse, BD Biosciences, USA) in 488 nm light (repeated three times). The flow cytometry analysis was completed by the Biomedical Analysis Center of the Shanghai University of Chinese Medicine.

The spreading of NIH3T3 cells was evaluated by SEM on the MT-MAA hydrogel at 1 d, 3 d, 5 d, and 7 d (each *n* = 8). The SEM protocols are presented in the supporting information.

#### Biodegradability test of the MT-MAA hydrogel in vivo, degradability of MTs in vitro, and pH value of the wound bed in the healing process

Subcutaneous implantation of MT-MAA hydrogel labeled by fluorescent dyes was used to test the in vivo biodegradation. Briefly, the hydrogel was prepared as described above, and flamma 675 (5 mg/ml, Vinylsulfone, BioActs, Korea) was added. The samples (2 ml) labeled by flamma 675 were transplanted subcutaneously into SD rats. These rats were assessed by daily in vivo imaging (*n* = 8), where the images were taken with 630 nm excitation on an IVIS Lumina LT (PerkinElmer, USA). Quantification of the fluorescence of the samples was analyzed using Living Image&#174 software. Furthermore, the biodegradability of the MT-MAA hydrogel was also evidenced by the decreased electrical conductivity of the hydrogel in vivo.

Depolymerization of MTs was assessed in different in vitro environments (pH (4–10), temperature (2–50 °C), CaCl_2_ (0–50 mM), each *n* = 8). MT solutions were prepared as described above. The MT solution (6 mg/ml) was transferred to 96-well plates, and the absorbance was measured at 340 nm at different time points using a plate reader (Fluoroskan FL, Thermo Scientific™. USA, repeated three times).

pH is an important variable in the micromovement of wounds over time, which can influence wound healing and conductivity of the dressing [36]. A pH meter (MP511, Thermo Scientific, USA) was used to measure the pH of the normal skin and the wound bed in the healing process (each *n* = 8, repeated three times).

### Cell behavior study and tube formation assay of the MT-MAA hydrogel/ES

#### MTT assay and scratch assay of NIH3T3 cells in the MT-MAA hydrogel/ES group

A scratch assay was conducted to explore the migration and angles of cells in the MT-MAA hydrogel/ES group, MT-MAA hydrogel group, MAA hydrogel/ES group, and control group (each *n* = 8) [18]. Briefly, ES was generated by a function generator (WAVE Factory 1952, NF Co., Yokohama, Japan) and carried out at 200 mV, with a 1MS pulse width at a frequency of 10 Hz. MAA hydrogel and MT-MAA hydrogel (500 µl) were sprayed into 6-well plates. NIH3T3 cells (1 × 10^5^ cells/well) were seeded on different groups and incubated at 37 °C for 1 d. The NIH3T3 cell monolayer was slightly scratched by a 500 µl pipette tip, and the detached cells were washed and removed by culture medium. The NIH3T3 cells of the MT-MAA hydrogel/ES group and MAA hydrogel/ES group were exposed to ES (30 min, 2 times/d) for 1 d. Images of wound gaps were captured by fluorescence microscopy. The distance of scratch gap and angles of NIH3T3 were analyzed by ImageJ 6.0.

The MTT assay was used to test the proliferation of NIH3T3 cells. The protocols are presented in the supporting information.

#### Evaluation of paracrine signaling in cells using RT‒PCR and ELISA

We tested the paracrine function of NIH3T3 cells in different groups using RT‒PCR and ELISA (each *n* = 8, repeated three times). To quantify the vascular endothelial growth factor (VEGF), transforming growth factor-β (TGF-β), and epidermal growth factor (EGF) gene expression in in-vitro culture at 3 d, mRNA from NIH3T3 cells of different samples was extracted and reverse-transcribed to cDNA using TaqPath™ 1-Step RT‒qPCR Master Mix (Thermo Scientific™. USA). RT-PCRs were conducted by CFX Opus Real-Time PCR Systems (Bio-Rad, California, USA). The primers for VEGF, TGF-β, and EGF are presented in the supporting information.

The conditioned culture supernatants of different samples were collected at 1 d, 3 d, 5 d, and 7 d. VEGF, TGF-β, and EGF of the different samples were tested with commercial kits (R&D Systems, USA) according to the manufacturer’s instructions (each *n* = 8, repeated three times).

#### Differentiation of MSCs

RT‒PCR was used to test the MSC differentiation induced by the MT-MAA hydrogel applied by ES, as described above. Calcium deposits of MSCs were shown by Alizarin Red S staining. The protocols for Red S staining and the primers for genes related to MSC differentiation are presented in the supporting information.

The migration and paracrine effects of MSCs induced by the MT-MAA hydrogel applied by ES were tested in NIH3T3 cells. The proliferation of MSCs induced by the MT-MAA hydrogel applied by ES was tested by BrdU experiment. The protocols of the BrdU experiment are presented in the supporting information.

#### Tube formation assay of ECs

MTs-MAA hydrogel and MAA hydrogel (500 µl) were coated onto cell culture dish (Cyagen, Guangzhou, China) and incubated at 37 °C for 20 min. ECs were seeded and cultured onto the dishes of the control group, MT-MAA hydrogel/ES group, MT-MAA hydrogel group and MAA hydrogel/ES group (each *n* = 10). The ES (carried out at 200mV, with a 1 MS pulse width at a frequency of 10 Hz) was applied for 30 min/d. Images were captured using a fluorescence microscope (TE2000-E, Nikon) after 3 d. The tube formation assay was analyzed using ImageJ 6.0.

### In vivo wound healing in a diabetic wound model

All animal experiments were conducted under protocols approved by the University of California Experimental Animal Ethics Committee, and every effort was made to minimize suffering with full protection of animal welfare. The experiments were performed on male C57BL/6J mice (6–8 weeks), which were provided by the animal center of the Shanghai University of Chinese Medicine.

Streptozotocin injection (i.p., 40 mg/kg, 5 d, Sigma‒Aldrich Shanghai, China) was used to develop type I diabetes in male C57BL/6J mice. Mice (blood glucose concentration > 10 mmol/L) were chosen as experimental animals. Under anesthesia, the hind limb of mice was depilated, and full-thickness excisional wounds on the hind limb were developed using a biopsy punch with a diameter of 3 mm by ligature. For detailed protocols, see reference [37]. The wound bed was grouped by treatment methods as follows: MT-MAA hydrogel/ES, MAA hydrogel/ES, MT-MAA hydrogel, and control (untreated) (each Group *n* = 20). ES was carried out at 200 mV, with a 1 MS pulse width at a frequency of 10 Hz (30 min/d).

Images of wounds were taken by camera on 0 d, 4 d, 7 d, and 14 d, and the wound closure rate were analyzed by ImageJ 6.0. The wound closure rate (%) was calculated as (current wound area/original wound area) %.

### Histological evaluation of re-epithelization, angiogenesis, infiltration of T/B immune cells and nerve repair

#### H&E staining and Masson staining were used to show the process of re-epithelization

The C57BL/6J mice were anesthetized, and their wound tissues were collected at scheduled time points. Samples were excised, fixed in 4% paraformaldehyde, and frozen in Tissue-Tek O.C.T. Section (8 μm thick, each *n* = 10). H&E staining was performed on the migrating epidermal tongue (MET) and re-epithelization. Briefly, slides were soaked in PBS for 5 min (repeated three times), stained with hematoxylin for 5 min, and washed in running water. Then, the slides were stained with 1% Eosin Y for 10 min and washed in running water. Images were captured with a fluorescence microscope (TE2000-E, Nikon) and analyzed using ImageJ 6.0. The protocols for Masson staining are provided in the supporting information.

#### Immunofluorescence staining was performed to assess angiogenesis and infiltration of T/B immune cells

The wound tissues were harvested and prepared on slides at 4 d. Then, immunofluorescence staining for DAPI and CD31 was used to evaluate angiogenesis. The slides were washed three times in PBS for 5 min and blocked with 3% BSA for 30 min at room temperature (each *n* = 10). The primary antibody (CD31, 1:200, Santa Cruz Corp., USA) was added and incubated at 4 °C overnight. Secondary antibody (1:800, Invitrogen Corp., Shanghai, China) was added for 1 h incubation at room temperature. After being washed with PBS, the cell nuclei were stained using DAPI.

The infiltration of T/B immune cells was studied by immunofluorescence staining in the slides of wound area at 4 d (each *n* = 10), and the procedures were performed as described above. Staining was performed for anti-CD79α for B cells (sc-20,064, 1:600, Santa Cruz Corp., USA), anti-CD3 (sc-20,047, 1:600, Santa Cruz Corp., USA) for T cells, and DAPI for cell nuclei. The images were captured with a fluorescence microscope in a blinded manner and analyzed using ImageJ 6.0.

#### Silver staining was performed for nerve fiber growth

Silver staining was conducted as previously reported [38]. The wound tissues were harvested and prepared into slides at 4 d and 7 d (each *n* = 10). The slides of samples were soaked in formaldehyde and washed three times in PBS for 5 min. Then, the slides were stained with silver nitrate solution (4% (w/v) for 1 h at 36.5 °C in the dark, deoxidized with 10% (v/v) formaldehyde, soaked in Tollens reagent for 10 min, and sealed. The images were taken by fluorescence microscopy in a blinded manner and analyzed using ImageJ 6.0, during which, the nerve fibers were marked and counted (repeated three times).

### Macrophage phenotype, growth factor expression and deposition during wound healing in vivo

#### Macrophage phenotype analysis by flow cytometry

Macrophages were isolated from wound area tissues at 4 d, the phenotype of which was analyzed by flow cytometry (each *n* = 8). Briefly, the wound area tissues were harvested, minced into small rectangular pieces, and soaked in digestion medium (8 mg/ml collagenase and 2.7 U/ml dispase II) at 37 °C for 20 min by a shaker. Then, excessive fetal bovine serum was added to stop digestion, and the cell suspension was harvested by a 50 μm cell strainer. After that, the cell suspension was centrifuged, resuspended in PBS containing 2% FBS, and double-stained with anti-CD11 (macrophage-associated marker, ab192343, Abcam, USA) and anti-CD206 (M2-associated marker, ab64693, Abcam, USA). Finally, flow cytometry was performed as described above.

#### RT‒PCR assay for growth factor expression

The wound tissues were harvested at 7 d (each *n* = 8). The tissues were minced into small rectangular pieces, and soaked in Buffer RLT (Qiagen, Guangzhou, China). mRNA was extracted using RNeasy Mini Kit (Qiagen, Guangzhou, China) according to the manufacturer’s instructions. Subsequently, ∼1 µg total RNA was reverse-transcribed to cDNA using TaqPath™ 1-Step RT‒qPCR Master Mix (Thermo Scientific™. USA). RT-PCRs were conducted by CFX Opus Real-Time PCR Systems (Bio-Rad, California, USA). The primers for VEGF, TGF-β, and EGF are presented in the supporting information.

#### Western blot assay for growth factor deposition

Western blotting was conducted to analyze the deposition of VEGF, EGF and TGF-β within the wound area at 7 d. The western blot protocols are provided in the supporting information.

### Antibacterial effect assay of MT-MAA hydrogel/ES

#### Antibacterial effect in vitro

E. coli and S. aureus were cultured in tryptic soy broth medium (Cyagen, Guangzhou, China). The MT-MAA hydrogel (1 ml) was coated onto a cell culture dish (Cyagen, Guangzhou, China). A bacterial suspension (1 ml 1 × 10^6^ CFU/ml) was added to the culture dish with ES (at 200 mV, with a 1 MS pulse width at a frequency of 10 Hz, 90 min). After 90 min, the bacteria were collected and double stained with PI/SYTO9 using a bacterial viability assay kit (ab189818, Abcam, USA). To analyze the antibacterial effect (each *n* = 8), flow cytometry was performed as described above (repeated three times).

#### Antibacterial effect in vivo

Full-thickness diabetic wounds were developed as described above. The wounds were covered by MT-MAA hydrogel applied by ES (with parameters as described above), with bacterial suspension (150 uL, 5 × 10^7^ CFU/ml) sprayed on the hydrogel (each *n* = 8). Images of the infected wound were taken at 1 d and 2 d in the control group and MT-MAA hydrogel/ES group, respectively. Purulent discharge and wound closure were observed as indicators of antibacterial effect in vivo.

### Statistical analysis

The experimental data were analyzed by SPSS 18.0, with values being expressed as the means ± standard errors. Each experiment was repeated at least three times. Statistical significance (*p < 0.05, **p < 0.01 and ^##^*p* < 0.05) was assessed by two-tailed tests.

## Results

### Characterization of MTs and the MT-MAA hydrogel (structure, conductivity, mechanical properties, biocompatibility, and biodegradability)

**Fig. 1:**
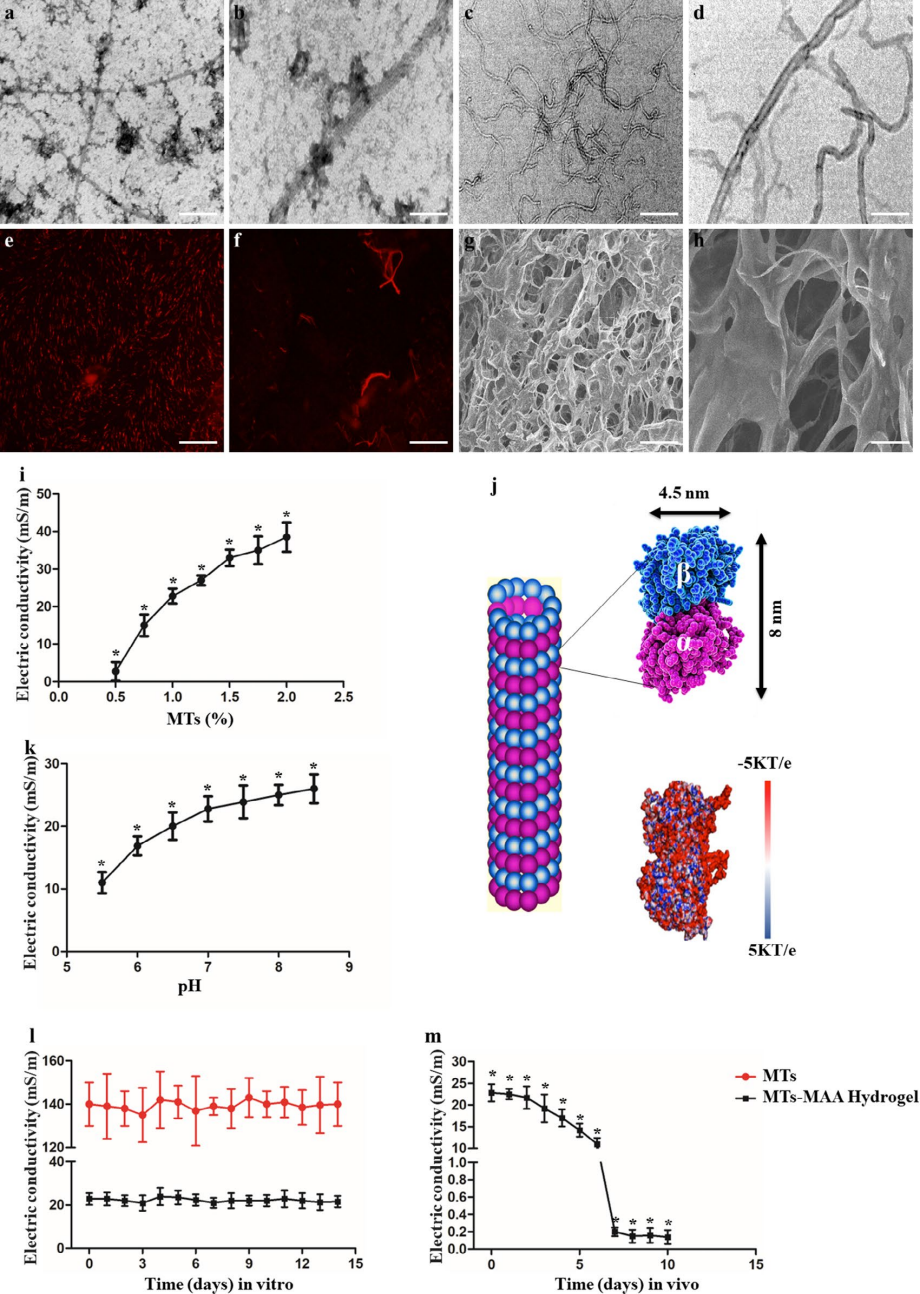
The structure and conductivity of MTs and the MT-MAA hydrogel. (**a**, **b**) Different magnifications of SEM images of MTs. (**c**, **d**) Different magnifications of TEM images of MTs. (**e**, **f**) Different magnifications of fluorescence microscopy images of MTs (HiLyte 488 dye-conjugated tubulin). (**g**, **h**) Different magnifications of SEM images of the MT-MAA hydrogel. (**i**) Conductivity of the MT-MAA hydrogel with 0.5-2 wt % MT. (**j**) Scheme of the structure of tubulin and electrostatic map of tubulin. (**k**) The conductivity of the MT-MAA hydrogel in an acidic environment in vitro. (**l**) MTs and the MT-MAA hydrogel maintained a steady conductivity within 14 d in vitro. (**m**) The conductivity of the MT-MAA hydrogel within 14 d in vivo. Scale bars represent 200 nm for (**a**, **c**), 50 nm for (**b**, **d**), 20 μm for (**e**), 1 μm for (**f**), 100 μm for (**g**), and 20 μm for (h). *p < 0.05, compared among all the groups

TEM and SEM of MTs showed a nanotube morphology (Fig. 1a and d). The MTs showed red linear fluorescence under fluorescence microscopy (Fig. 1e and f). HiLyte 488 dye-conjugated tubulin). MTs consist of 13 protofilaments, which are assembled by tubulin monomers in a head-to-tail manner. The outer diameter of the MT is 25 nm, and the lumen diameter of the MT is 15 nm [39]. SEM demonstrated that the MT-MAA hydrogel had a nanofibrous and microporous structure (Fig. 1g and h).

By electroorientation method, the conductivity of MTs was measured to be 0.14 S/m in PBS solution, and MTs maintained a steady conductivity within 14 d in vitro (Fig. 1l). Moreover, the conductivity of MTs was increased in the presence of monovalent cations, but significantly impaired by divalent cations and the acidic environment (Fig. S1). It is well known that electrostatic polarity is a characteristic of MTs, mainly along the vertical axis. MTs were aligned by E-fields (Fig. S1). The structural scheme and the electrostatic map of tubulin are shown under physiological pH (Fig. 1j) [20].

As shown in Fig. 1i, the MAA hydrogel was almost nonconductive, while the conductivity of the MT-MAA hydrogel increased from 2.73 × 10^− 3^ to 3.85 × 10^− 2^ S/m when the MT content was increased from 0.5 to 2 wt %, indicating a favorable conductivity of the MT-MAA hydrogel. Conductive wound dressings with a conductivity similar to that of human skin exhibit a great potential in the enhancement of wound healing, especially for chronic wounds [40]. Skin has a conductivity value from 1 × 10^− 5^ to 2.6 × 10^− 1^ S/m, depending on its components [19]. We used 1% (MT content wt%) MT-MAA hydrogel as the experimental condition in the following experiments, mimicking the conductivity of skin. The MT-MAA hydrogel maintained a steady conductivity within 14 d in vitro (Fig. 1l), which was weakened over time in vivo, especially after 7 d (Fig. 1m). Moreover, the conductivity of the MT-MAA hydrogel was significantly impaired by the divalent cations and acidic environment (Fig. 1k, Fig. S2). pH is an 3important variable in the micromovement of wounds over time, which could influence wound healing and the conductivity of dressings [36]. Our results showed that the pH of normal skin was 5.9 ± 0.8, which increased to 8.1 ± 0.4 upon injury. The wounds recovered from the acidic microenvironment during the healing process (Fig. S3), while the acidic microenvironment impaired the conductivity of the MT-MAA hydrogel in vivo.

**Fig. 2:**
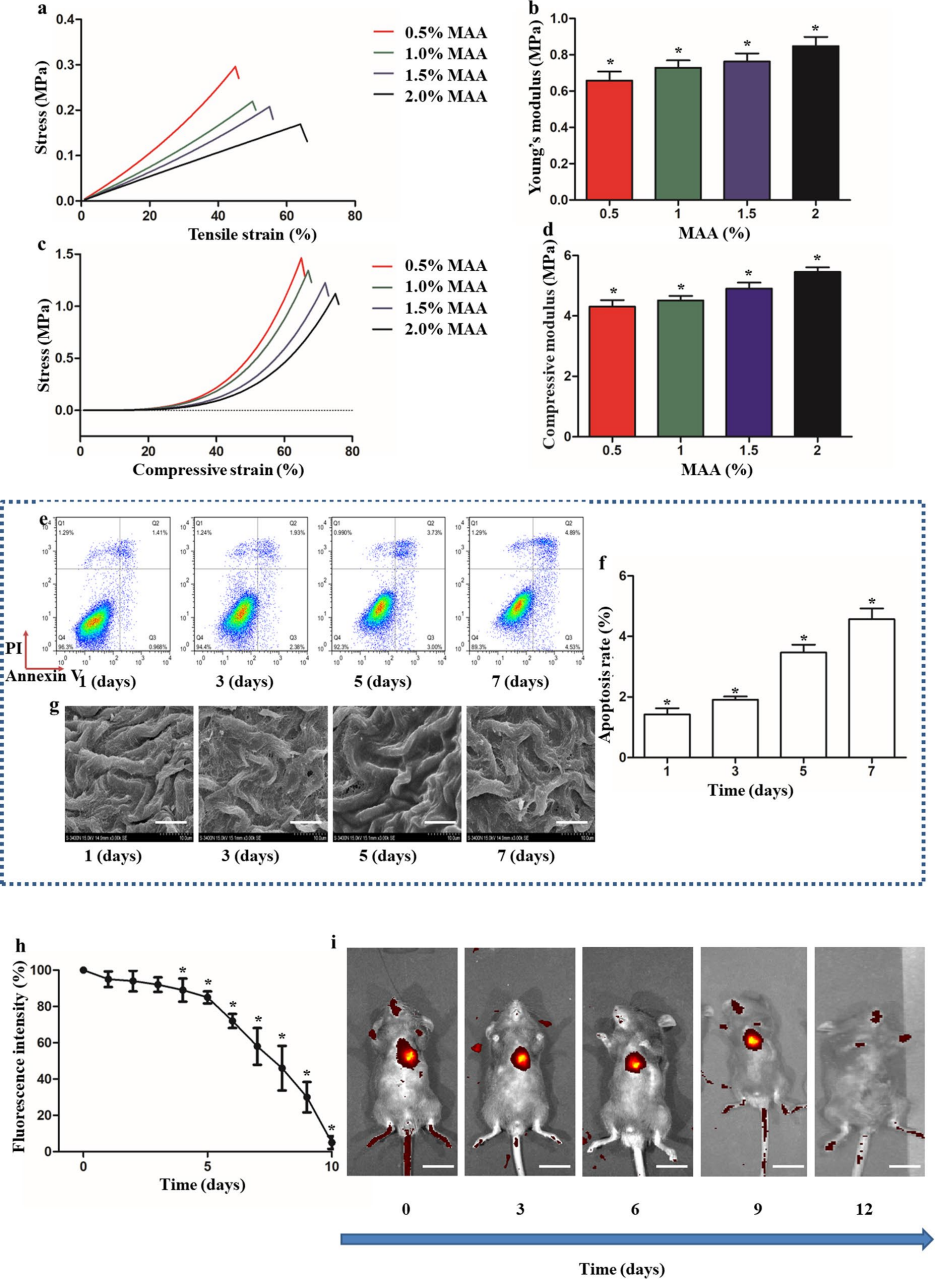
The mechanical properties, biocompatibility, and biodegradability of the MT-MAA hydrogel. (**a**, **b**) Tensile stress–strain curves and Young’s modulus of the MT-MAA hydrogel (0.5 to 2 wt % MAA). (**c**, **d**) Compressive stress–strain curves and compressive modulus of the MT-MAA hydrogel (0.5 to 2 wt % MAA). (**e**, **f**) The apoptosis rate of NIH3T3 cells at 1 d, 3 d, 5 d, and 7 d on the MT-MAA hydrogel. (**g**) SEM images of spreading NIH3T3 cells at 1 d, 3 d, 5 d, and 7 d (scale bar = 10 μm). (**h**, **i**) The biodegradability of the MT-MAA hydrogel in vivo (scale bar = 6.5 cm). *p < 0.05, compared among all the groups

In addition to conductivity, favorable mechanical properties are another important factor for wound dressing, which is essential to angiogenesis and the function of infiltrating cells [41]. The results of the stress–strain curves and mechanical modulus of the MT-MAA hydrogel are shown in Fig. 2a and d. The hydrogel gradually increased in Young’s modulus and compressive modulus when the content of MAA was raised from 0.5 wt% to 2 wt%. The maximum compressive strain and the tension strain of different groups are shown in Table S2. The 2% MAA hydrogel was chosen as the experimental condition in the following experiments.

The biocompatibility of the MT**-**MAA hydrogel was studied by apoptosis of NIH3T3 cells using flow cytometry. The levels of apoptosis in the MT**-**MAA hydrogel group at 1 d, 3 d, 5 d, and 7 d were 1.41%, 1.93%, 3.73% and 4.89%, respectively (Fig. 2e and f). NIH3T3 cells were seeded in the MT-MAA hydrogel. At 1 d, 3 d, 5 d, and 7 d, NIH3T3 cells were spread and remained in the MT-MAA hydrogel, as shown by SEM (Fig. 2g). These results demonstrated that the MT-MAA hydrogel can promote cell apoptosis and show good biocompatibility.

The biodegradability of the MT-MAA hydrogel (red fluorescence labeled) was assessed by in vivo imaging on a daily basis. The MT-MAA hydrogel showed an obvious loss in red fluorescence resulting from biodegradation after 10 d (Fig. 2h and i). Depolymerization and polymerization of MTs were assessed using the CytoDYNAMIX Screen kit (BK006P; Cytoskeleton Inc., Denver, CO, USA) at different time points. In vitro, MTs maintained an excellent station of polymerization within 14 d (Fig. S4), and the station of polymerization was changed by pH, temperature, and Ca^2+^ concentration (Fig. S4). Similar results were found using in situ immunofluorescence labeling of MTs under various environments (pH, temperature, and cations) (Fig. S4). High concentrations of small divalent cations (Ca^2+^, above concentrations of 40 mM) were found to induce MTs to depolymerize. MTs were depolymerized below pH 5.0 and above pH 8.5, and below 4℃ and above 40℃.

### Enhancement of aligned migration, proliferation, paracrine of cells as well as tube formation were induced by the MT-MAA hydrogel applied by ES in vitro

NIH3T3 cells were used for this study, which play a crucial role in wound repair [42].

**Fig. 3:**
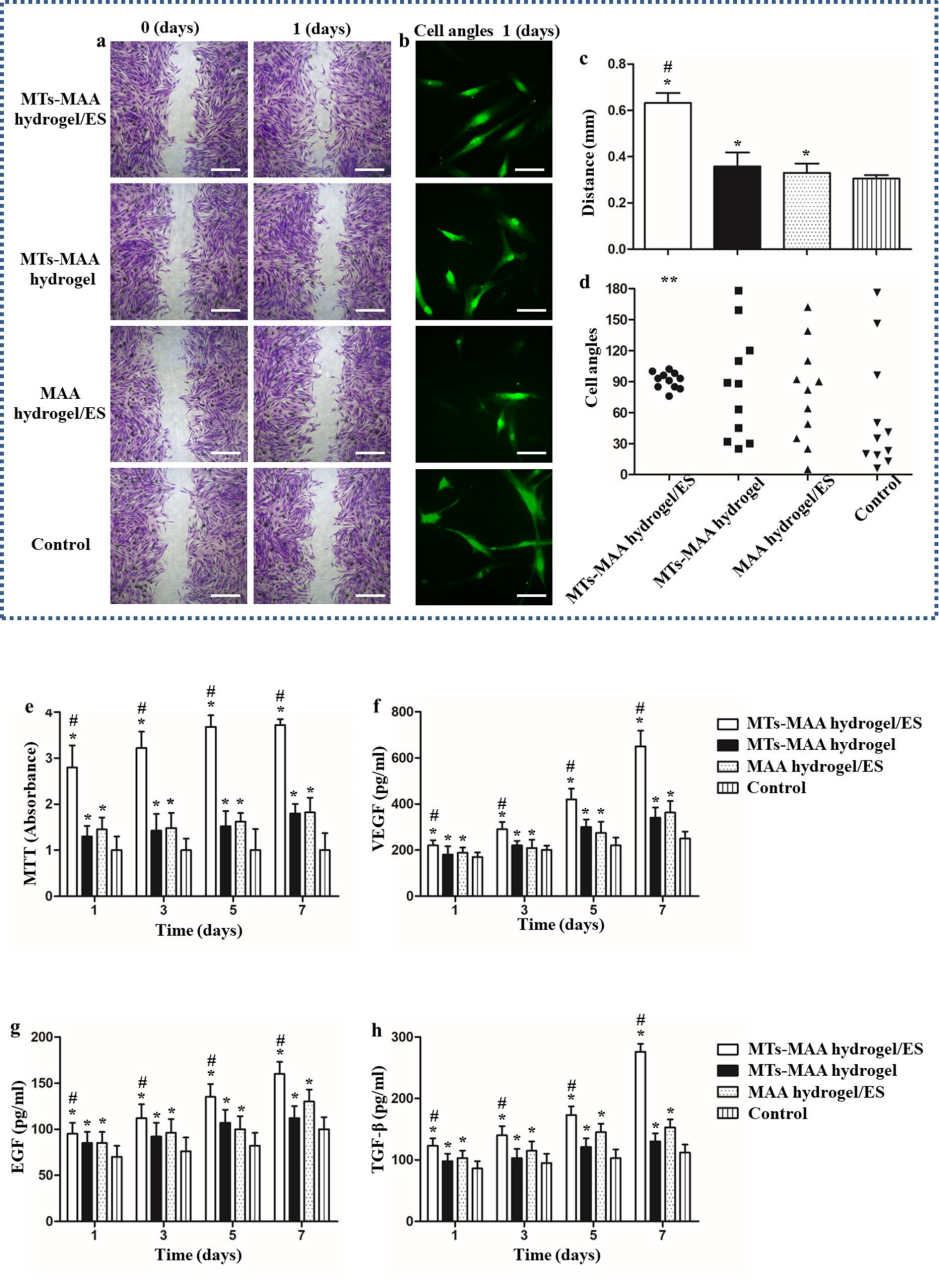
Enhancement of aligned migration, proliferation, and paracrine signaling of cells in MT-MAA hydrogels treated with ES in vitro. (**a**, **b**) Microscopic images of the scratch assay. (**c**, **d**) Quantification of migration distance and angles at 1 d in different groups. (**e**) The MTT assay at different time points. (**f**–**h**) The secretion of growth factors at different time points. Scale bars represent 20 μm for (**a**) and 5 μm for (**b**). *p < 0.05, **p < 0.01, compared with the control group. ^#^*p* < 0.05, compared with the MT-MAA hydrogel group and MAA hydrogel/ES group

A scratch assay was conducted to show the migration of NIH3T3 cells by 2D cell culture coated by the MT-MAA hydrogel applied by ES (Fig. 3a and b). The migration distances in the MT-MAA hydrogel/ES group, MT-MAA hydrogel group, MAA hydrogel/ES group, and control group at 1 d were 0.62 ± 0.05 mm, 0.37 ± 0.07 mm, 0.36 ± 0.03 mm, and 0.30 ± 0.02 mm, respectively (Fig. 3c). The results suggested that the MT-MAA hydrogel/ES group had significantly more efficient migration than the other groups (the MT-MAA hydrogel group, MAA hydrogel/ES group, and control group). The angles of NIH3T3 cells (green labeled) were quantitatively analyzed in different groups. The migration of the MT-MAA hydrogel/ES group showed alignment, while the other groups showed nonalignment (Fig. 3b and d).

MTT assay was used to study cell proliferation by absorbance at 450 nm in different groups. The results showed that the MT-MAA hydrogel/ES group had significant proliferation at different time points compared to the other groups. This confirmed that ES could improve cell proliferation via the conductivity of the MT-MAA hydrogel. Meanwhile, the MT-MAA hydrogel group and MAA hydrogel/ES group had obvious proliferation compared to the control group, which suggested that the MAA hydrogel can promote the proliferation of NIH3T3 cells (Fig. 3e).

The cellular metabolism of NIH3T3 cells in different groups was tested. VEGF, TGF-β, and EGF were tested in the supernatant of the cell culture medium by ELISA. The secretion of the three growth factors was significantly increased in the MT-MAA/ES group at different time points compared to the other groups (Fig. 3f and h). The gene expression of growth factors showed a similar result as the ELISA (Fig. S5).

Angiogenesis is a vital process in wound repair. The tube formation experiment was conducted in vitro to assay different groups by ECs. There was a significant improvement in tube formation in the MT-MAA hydrogel/ES group compared to the other groups at 3 d. By quantitative analysis, the relative tube length(%) in the MT-MAA hydrogel/ES group, MT-MAA hydrogel group, and MAA hydrogel/ES group was 153.5%, 105.7%, and 110.3%, respectively (Fig. S6).

Moreover, the MT-MAA hydrogel, as a tissue engineering scaffold seeded with stem cells, was tested in chronic wounds applied by ES. MSCs which are commonly used for skins tissue engineering were chosen as the stem cell line in this study [43]. MSCs can differentiate into many special cell types. The MSC differentiation potential was revealed by RT‒PCR and Red S staining. In the MT-MAA hydrogel/ES group, the results showed that ES improved osteogenic gene expression in MSCs. Calcium deposition became obvious in the MSCs of the MT-MAA hydrogel/ES group at 14 d compared to the other groups (Fig. S7). Moreover, the effects of the MT-MAA hydrogel/ES on the behaviors of MSCs were shown in Fig. S8–S10, and the results indicated improvement in aligned migration, proliferation and paracrine signaling of MSCs, similar to NIH3T3 cells.

### A full-thickness diabetic wound mode revealed rapid wound closure, angiogenesis, and re-epithelialization using MT-MAA hydrogel applied by ES

**Fig. 4:**
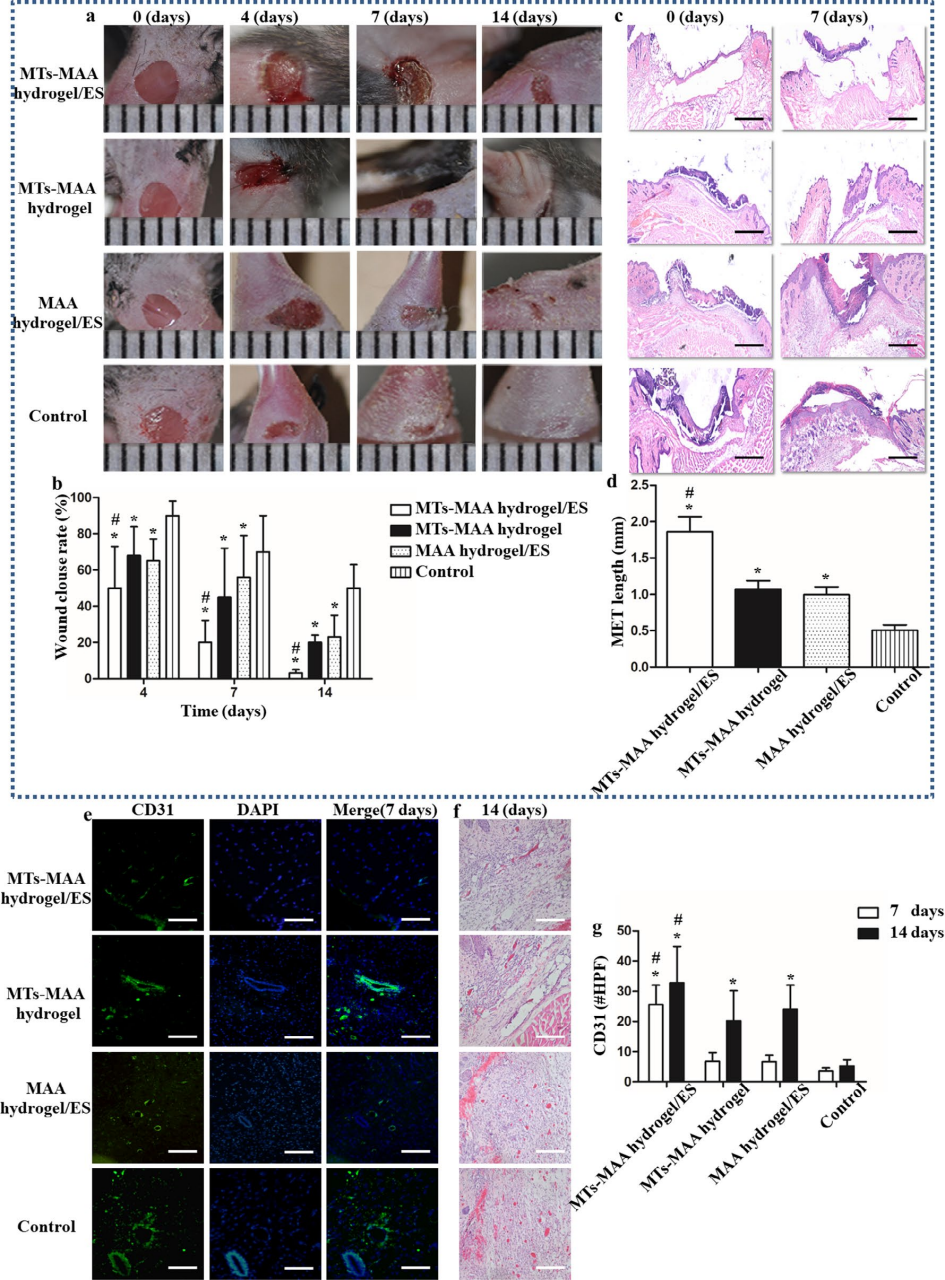
A Full-thickness diabetic wound model revealed rapid wound closure, angiogenesis, and re-epithelialization using the MT-MAA hydrogel applied by ES. (**a**) Images of wounds at 0 d, 4 d, 7 d, and 14 d. (**b**) Wound clouse in different groups. (**c**) H&E staining of granulation tissue at 4 d (scale bar = 1 mm). (**d**) Quantification of MET length at 4 d. (**e**) The staining of CD31-positive microvessels of wounds at 7 d. (green) CD 31; (blue) DAPI; (scale bar = 100 μm). (**f**) H&E staining of wounds at 14 d (scale bar = 100 μm). (**g**) Quantitative analysis of CD31-positive microvessels. *p < 0.05, compared with the control group. ^#^*p* < 0.05, compared with the MT-MAA hydrogel group and MAA hydrogel/ES group

To determine the wound closure efficacy of the MT-MAA hydrogel/ES in vivo, a full-thickness diabetic wound model was created on the legs of C57 mice, where the prepared dressings were applied. Figure 4 shows images of the wounds in the control group, MT-MAA hydrogel group, MAA hydrogel/ES group, and MT-MAA hydrogel/ES group at 0 d, 4 d, 7 d, and 14 d. The results showed that the MT-MAA hydrogel/ES group received the best wound-healing effect, with rapid wound closure within 7 d. No obvious signs of wound infection were observed in dressing-covered wounds within 14 d (Fig. 4a). The epidermal tissue grew to the wound area center, promoting the healing of diabetic wounds in the MT-MAA hydrogel/ES group. The MT-MAA hydrogel/ES group showed faster wound regeneration than the other groups (Fig. 4b).

To further study the effect of MT-MAA hydrogel/ES on the wound healing process, hematoxylin & eosin (H&E) staining was conducted to assay the healed tissues. The results revealed that the MT-MAA hydrogel/ES group had more epidermal tissue migration and growth than the other groups in the wound bed at 7 d (Fig. 4c). The migrating epidermal tongue (MET) length of MTs-MAA hydrogel/ES group were 1.82 ± 0.3 mm; 0.50 ± 0.3 mm, control group; 1.14 ± 0.2 mm, MTs-MAA hydrogel group; and 1.07 ± 0.2, MAA hydrogel/ES group (Fig. 4d).

Improving angiogenesis in chronic wounds has become the focus for wound repair, especially among diabetic patients. We confirmed wound angiogenesis by CD31 staining and H&E staining. The CD31-positive (green) tissues of different groups at 7 d are shown in Fig. 4e, and it was indicated that the MT-MAA hydrogel/ES group had significant angiogenesis compared to the other groups. Quantitative analysis of the vascular density of the wound area in the MTs-MAA hydrogel/ES group were 25.6 ± 5.4; 3.6 ± 0.3, control group; 6.8 ± 1.3, MTs-MAA hydrogel group; and 6.7 ± 1.7 (#/HPF), MAA hydrogel/ES group, respectively (Fig. 4g). The obvious angiogenesis of the MT-MAA hydrogel group and MAA hydrogel group was also observed at 14 d (Fig. 4f and g).

Finally, the MT-MAA hydrogel was tested in chronic wounds as a tissue engineering scaffold seeded with MSCs applied by ES. The wound healing process were under observation within 7 d. The results showed that the MT-MAA hydrogel seeded with MSCs applied by ES promoted rapid wound healing within 7 d (Fig. S11). Tissue remodeling processes and angiogenesis were assessed in vivo using H&E (Fig. S11), Masson staining (Fig. S11), and CD 31 staining (Fig. S12).

### Limitation of the inflammation phase and increase in growth factors in the MT-MAA hydrogel/ES group during wound healing

**Fig. 5:**
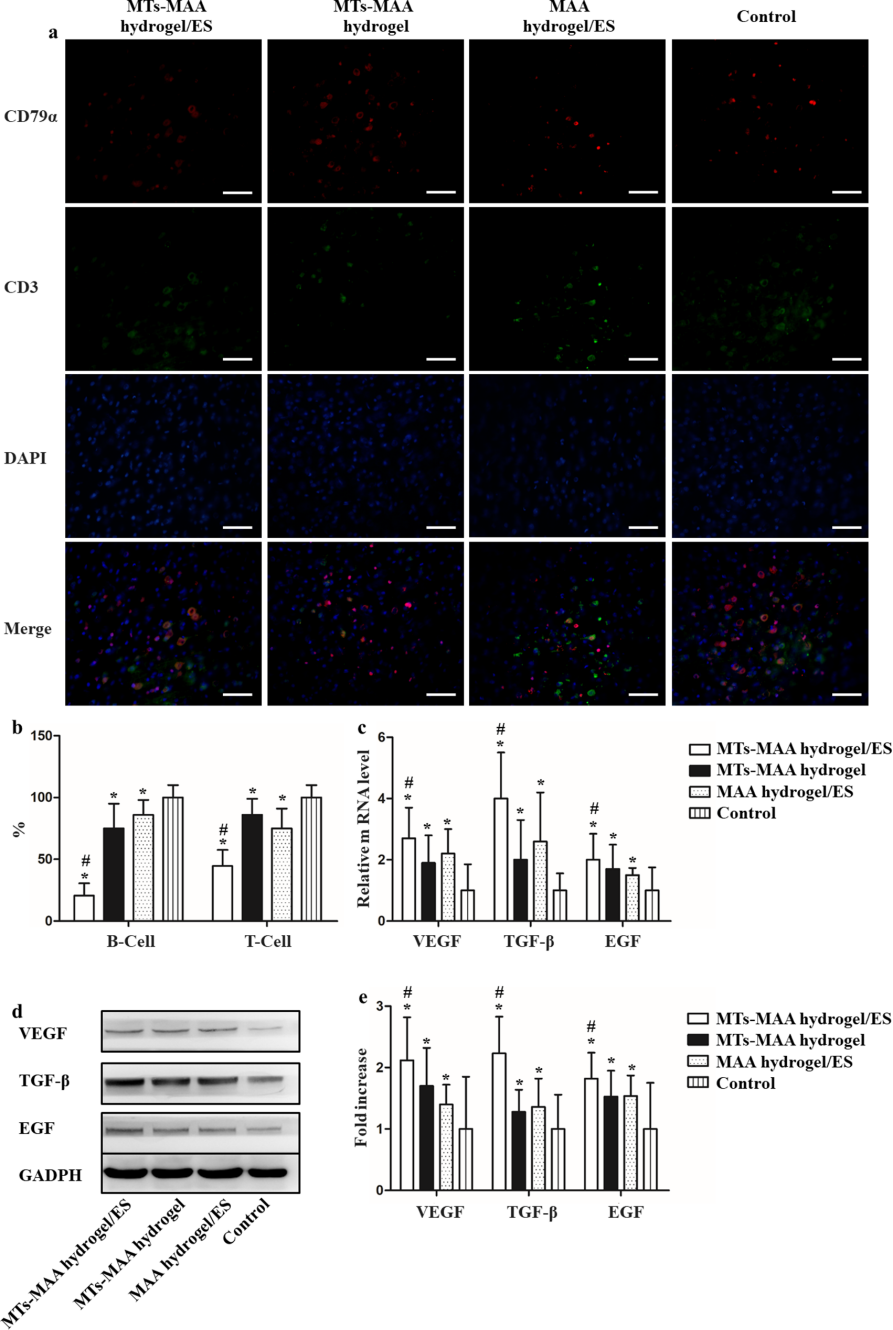
Limitation of T/B-cell infiltration and increase in growth factors in the MT-MAA hydrogel/ES group during wound healing. (**a**) The staining of CD79α and CD3 for T/B-cell infiltration. (red) CD79α; (green) CD3; (blue) DAPI; (scale bar = 20 μm). (**b**) Quantification of T/B-cell infiltration in different groups. (**c**) Gene expression of growth factors in wounds at 7 d. (**d**, **e**) The deposit of growth factors in wounds at 7 d using western blotting. *p < 0.05, compared with the control group. ^#^*p* < 0.05, compared with the MT-MAA hydrogel group and MAA hydrogel/ES group

Infiltration of T/B immune cells was studied by immunofluorescence staining (CD79α for B cells; CD3 for T cells and DAPI staining for cell nuclei) in the wound area (Fig. 5a). The results showed that the MT-MAA hydrogel/ES group had less T/B immune cell infiltration at 4 d than the other groups. For the MT-MAA hydrogel/ES group, the infiltration of T cells was decreased by 79.5%, and B cells were decreased by 55.4% using quantitative analysis compared to the control group. The MT-MAA hydrogel group and MAA hydrogel/ES group also had less T/B immune cell infiltration than the control group. No significant difference was observed between the MT-MAA hydrogel group and the MAA hydrogel/ES group (Fig. 5b).

Macrophages are important inflammatory cells, and the macrophage phenotypes play an important role in wound healing [31]. Compared to the other groups, a significant impact of phenotype was observed in the MT-MAA hydrogel/ES group. The results confirmed the transitioning of the macrophage phenotype from pro-inflammatory/M1 state toward pro-repair/M2 state in the MT-MAA hydrogel/ES group. Quantitative analysis revealed that infiltration of pro-repair/M2 cells increased by 170.5% compared to other groups. The MT-MAA hydrogel group and MAA hydrogel/ES group also had higher level of pro-repair/M2 activity than the control group. There was no significant difference between the MT-MAA hydrogel group and the MAA hydrogel/ES group, which was discernible at 4 d (Fig. S13). These results revealed that the MT-MAA hydrogel/ES group had more remodeling phases by inducing an inflammation phase.

Three growth factors (VEGF, TGF-β and EGF) related to wound healing were studied in this research. The gene expression of VEGF, TGF**-**β and EGF was measured using RT‒PCR. The MT-MAA hydrogel/ES group expressed the highest mRNA levels of VEGF, TGF**-**β and EGF compared to the other groups at 7 d (Fig. 5c). Moreover, western blotting was used to show the deposition of VEGF, TGF**-**β and EGF protein in the wound area. The MT-MAA hydrogel/ES group had the most amount of deposition of VEGF, TGF-β and EGF protein compared to the other groups at 7 d (Fig. 5d and e).

### Improvement of nerve fiber growth and antibacterial efficiency in the MT-MAA hydrogel under ES

**Fig. 6:**
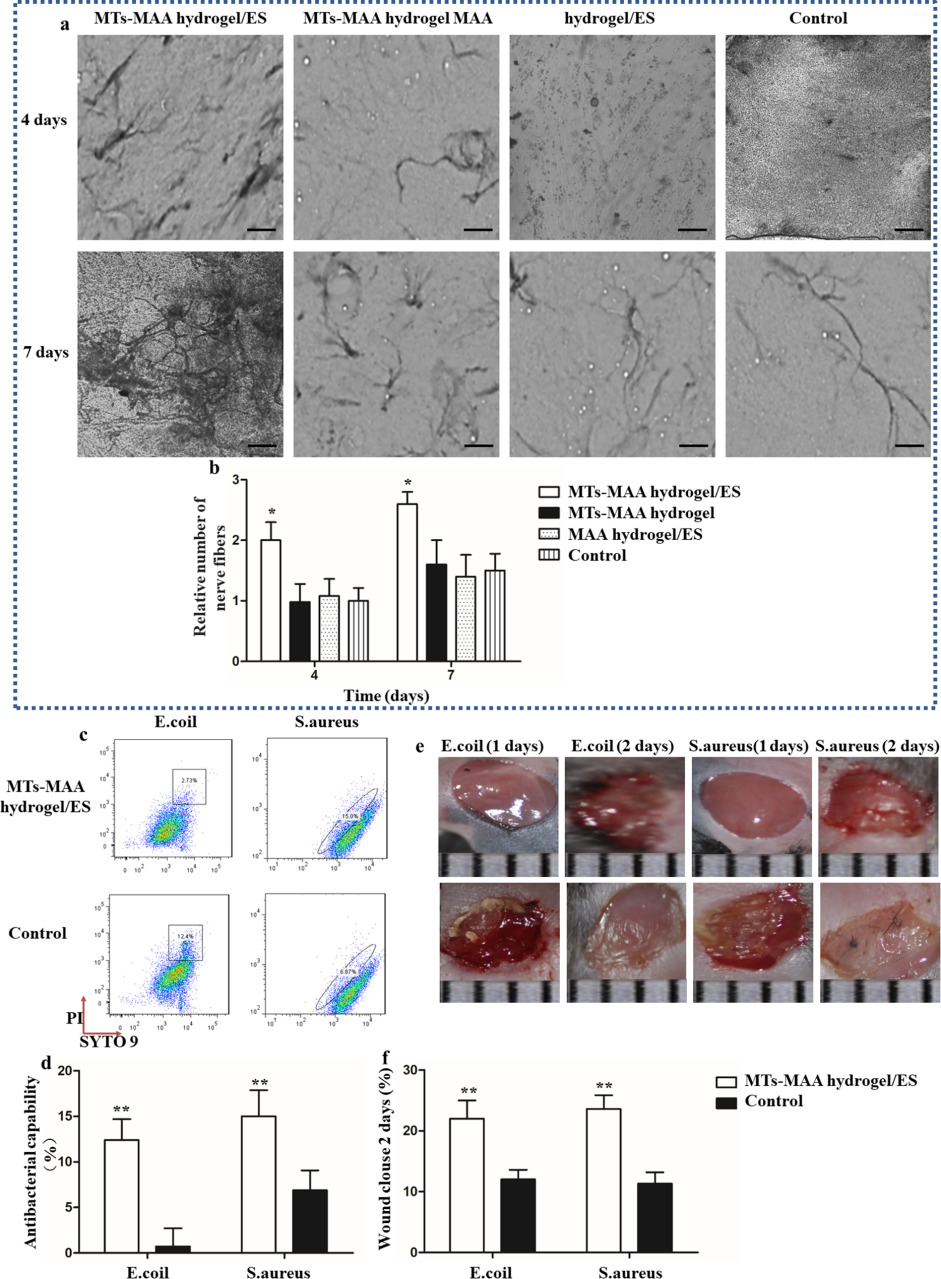
Improvement of nerve fiber growth and antibacterial efficiency in the MT-MAA hydrogel/ES group. (**a**) Silver staining for nerve fiber growth at 4 d and 7 d (scale bar = 50 μm). (**b**) Quantification of nerve fiber number in different groups. (**c**) Flow cytometry analysis of antibacterial efficiency in different groups. (**e**) Images of infected wounds in different groups at 1 and 2 d. (**f**) Wound clouse of infected wounds in different groups. *p < 0.05, **p < 0.01, compared among all the groups

Nerve regeneration and antibacterial efficiency are vital factors for wound repair, especially for diabetic and refractory wounds [44]. Silver staining was performed for nerve fiber growth. As shown in Fig. 6a, the MT**-**MAA hydrogel/ES group had significant nerve fiber growth of 2.6 ± 0.4 at 7 d (2.0 ± 0.3, 4 d) compared to the other groups. There was no significant difference among the other groups. Furthermore, the degree of nerve fiber growth was enhanced in each group (7 d vs. 4 d) (Fig. 6b). In the group of MT**-**MAA hydrogel/ES seeded with MSCs, the number of nerve fibers was 3.3 ± 0.7 at 7 d and 2.1 ± 0.5 at 4 d (Fig. S14).

The results suggested that the MT-MAA hydrogel/ES group had significant in vitro antibacterial efficiency (MT-MAA hydrogel/ES group: E. coli, 12.4% and S. aureus, 15.0%; control group: E. coli, 2.73% and S. aureus, 6.87) (Fig. 6c and d). In vivo, images of the infected wound were taken at 1 d and 2 d in the control group and MT-MAA hydrogel/ES group. As shown in Fig. 6e, infection was observable in the control group, but non-visible in the MT-MAA hydrogel/ES group. The MT-MAA hydrogel/ES group had better wound closure with E. coli and S. aureus (22.04 ± 3.32%, 23.60 ± 2.31%) at 2 d compared to the control group (12.0 ± 1.6%, 11.3 ± 1.9%) (Fig. 6f).

## Discussion

The lack of a suitable conductive biomaterial limits the clinical application of electrical stimulation (ES) in chronic wounds [14]. Therefore, developing a clinical conductive dressing has become a popular research topic. In this study, we developed a novel conductive microtubule (MT) hydrogel based on biological electrical wires of the body.

MTs were polymerized by α- and β-tubulin monomers in vitro. SEM, TEM, and fluorescence microscopy showed a hollow cylindrical structure of MTs with an outer diameter of 25 nm and a lumen diameter of 15 nm. Previous studies have shown the same structure of MTs [34]. The conductivity of MTs is 0.14 S/m in similar wound microenvironments and are influenced by various factors (pH and ion species, etc.), as indicated by the in vitro experiments. The conductivity of MTs was highest in the presence of monovelent ions compared to divalent and trivalent ions. Divalent and trivalent ions have stronger ionic forces with MTs, which would decrease the ion flow [24]. The conductivity of MTs was impaired in an alkaline environment while the increased pH value of the solution can increase the net charges of tubulin, resulting in enhanced conductivity of MTs [24]. When the C-terminus of tubulin is enzymatically cleaved by subtilisin, the conductivity is reduced to 60%, which plays an important role in MT-based charge transport [45]. MTs exhibit the properties of a responsive-conductive biomaterial in different environments.

Although the conductivity of the MT-MAA hydrogel increased from 2.83 × 10^− 3^ to 3.85 × 10^− 2^ S/m (0.5-2%, MT wt%) in vitro, it was essential to find out whether the biodegradation and wound microenvironment would influence the conductivity of the MT-MAA hydrogel in vivo. Suitable biodegradation is a key factor in maintaining the conductivity of MT-MAA hydrogels and improving wound healing. The in vivo results demonstrated that the MT-MAA hydrogel shows a loss in fluorescence resulting from hydrogel degradation at 10 d, which provides an appropriate degradation time for wound regeneration. The cross-linking strength and the degree of methacrylate of the MAA hydrogel are important factors for biodegradation [29, 46]. Meanwhile, the concentration of Ca^2+^, exudate, enzymes, and infiltrating cells of wounds have a significant influence on biodegradation in vivo [29, 31].

The conductivity of the MT-MAA hydrogel was significantly weakened after 7 d in vivo. The conductivity decreased faster than hydrogel biodegradation as a result of the acidic environment caused by wound repair. Both previous reports and our results show that skin is naturally acidic with a pH value of 5.5 ± 0.7, while the wound bed is alkaline (pH: 7 to 8) [36]. During the reconstruction of the skin tissue in chronic wounds, recovering the acidic environment of the wound can accelerate wound healing. The acidic environment decreased the conductivity of MTs, which caused the conductivity to decrease faster than hydrogel biodegradation. In vitro experimental results showed that the conductivity of MTs was impaired with the increase of the pH of the solution, and MTs were depolymerized below pH 5.0 during wound repair, similar to the results produced by previous reports [24]. The biodegradation of the MT**-**MAA hydrogel induced an increase in free calcium ions, which was another reason that conductivity decreased faster than hydrogel biodegradation. Studies have revealed that Ca^2+^ can significantly decrease the electrical conductivity of MTs [47]. MTs maintained a steady station of polymerization and conductivity within 14 d, and the polymerization and depolymerization of MTs were altered by temperature, pH, and divalent cations in vitro. Previous studies have shown that high concentrations of small divalent cations (Ca^2+^, Ba^2+^, and Sr^2+^) induce MTs to assemble into loose 2D bundles (above concentrations of 40 mM, 60 mM, and 60 mM, respectively) [48]. MTs respond differently in different environments, and MTs-based responsive biomaterials can be applied in other fields (e.g., responsive drug delivery systems).

The MT-MAA hydrogel did not have a significant influence on cell viability over a dynamic period in vitro. The depolymerization product of MTs is tubulin, which is widely expressed in cells and has excellent biocompatibility [20, 48]. MAA is commonly used in tissue engineering and has good biocompatibility. Alginate oligosaccharide sodium (AOS) biodegraded from MAA has been carefully studied and used in wound repair [29, 46, 49]. Compared to other conductive biomaterials, the MT-MAA hydrogel has favorable biocompatibility. It may be a good strategy to screen a suitable conductive biomaterial from an inherent structure derived from the body based on the electrical activity of the human body. ES could enhance the migration and proliferation of NIH3T3 cells by the conductivity of the MT-MAA hydrogel in vitro, and similar results were observed in ES reports [50]. Cell alignment was observed in the MT-MAA hydrogel/ES group, which was a result of cytoskeleton reorientation through Wnt/β-catenin pathways induced by ES [51]. The MTs were oriented parallel to the field line in vitro because of the dipole moment along the MT long axes, which may be another reason for the alignment of migration [20]. Enhancement of migration and proliferation improved re-epithelialization in the MT-MAA hydrogel/ES group. In an in vivo study, a full-thickness diabetic wound model revealed rapid wound closure within 7 d. The hydrogel maintained a suitable electrical conductivity within 7 d, proving to be a steady conductive dressing for wound regeneration.

According to previous literatures, ES can improve wound healing by angiogenesis [52] and extracellular matrix remodeling., etc [53]. . . In vitro, MT-MAA hydrogel/ES could promote tube formation of ECs. Previous studies have shown that ES induces angiogenesis by stimulating vessel tube formation via the induction of angiogenic signaling pathways [54]. The angiogenesis of the MT-MAA hydrogel/ES group was enhanced at 7 d compared to that of the other groups in vivo. The hydrogel maintained a steady state and good electrical conductivity within 7 d. The ES played an important role in angiogenesis by the conductivity of MT-MAA hydrogel within 7 d. The angiogenesis of the MT-MAA hydrogel and MAA hydrogel groups was also observed at 14 d, and the conductivity of the dressing was significantly weakened at 7 d. The angiogenesis may be induced by AOS biodegraded from MAA [29], as AOS has a significant ability of angiogenesis in tissue regeneration [29]. Another explanation may be that the growth factors secreted by infiltrating cells promote angiogenesis [55]. In this study, levels of TGF-β, EGF, and VEGF (three typical growth factors) expression and deposition in the wound area of MT-MAA hydrogel/ES group at 7d were found to be higher than those of other groups.

Conductive dressings applied by ES could enhance phagocytosis and antibacterial activity and shorten the inflammatory phase to accelerate wound healing, especially in infected wounds [11, 17, 53]. The results showed that the MT-MAA hydrogel/ES group had less T/B immune cell infiltration, transitioning the macrophage phenotype from pro-inflammatory/M1 state toward pro-repair/M2 state at 4 d. Also, it was found that the MT-MAA hydrogel/ES shortened the inflammation phase, shifting quickly to the proliferative and remodeling phases. The MT-MAA hydrogel/ES maintained good conductivity within 7 d, providing a conductive dressing for controlling inflammation. Limited inflammation was observed at 4 d in the MAA hydrogel group, possibly because that MAA limits the inflammation and bacterial infections of wounds. MAA can exert anti-inflammatory and immunomodulatory effects by inducing nitric oxide (NO), TNF-α, and NF-KB release from macrophages and antibacterial activity (S. aureus, E. coli, etc.) [49]. The present experiment found that MAA is an ideal hydrogel scaffold to load MTs, which exhibits suitable biodegradation and biocompatibility, improves angiogenesis, limits inflammation phase, and promotes antibacterial effects.

Nerve repair can enhance chronic wound healing by various pathways, especially in diabetic wounds [44]. ES prominently enhances nerve fiber growth via conductive biomaterials by increasing the expression of BDNF neurotrophies and outgrowth of axons [56]. Obvious nerve fiber growth was observed in the MT-MAA hydrogel/ES group. Previous studies have revealed that the alignment of conductive biomaterials is conducive to improving nerve regeneration [9]. MTs can be aligned by an electric field because of the dipole moment induced along MTs [20, 21, 39], which was another explanation for the obvious nerve fiber growth in the MT-MAA hydrogel/ES group. These results support the idea that MTs-based biomaterials may be promising therapies for nerve regeneration.

The MT-MAA hydrogel as a tissue engineering scaffold was tested in chronic wounds. The results showed that the MT-MAA hydrogel seeded with MSCs applied by ES can promote wound healing within 7 d. In the MT-MAA hydrogel/ES group, osteogenic differentiation of MSCs was observed at 14 d in vitro, as previously reported [57]. There was no obvious osteogenic differentiation in histopathologic assessment at 7 d. ES application within a limited time period (7 d) could finish wound closure without leading to significant osteogenic differentiation of MSCs. Osteogenic differentiation of MSCs may be used for bone tissue engineering with MT-MAA hydrogel/ES. The accelerated wound healing may be explained by the regenerative capacity of MSCs and ES [43].

## Conclusions

Chronic wounds are a common health burden, especially among diabetic and paralyzed patients. There has been no effective method to promote wound healing. Electrical stimulation (ES) is considered a promising therapy for chronic wounds via conductive dressing. However, the lack of a clinically suitable conductive dressing is a serious challenge. In this study, we developed a novel conductive hydrogel for electrical stimulation (ES) in chronic wound repair based on the biological electrical wire properties of microtubules (MTs). The results showed that the MT-MAA hydrogel has favorable conductivity, biodegradability, and biocompatibility, and exhibited an elevated secretion of growth factors with enhanced cell proliferation and migration ability in response to ES in vitro. In vivo, the MT-MAA hydrogel applied by ES revealed rapid wound closure, promoted angiogenesis and nerve growth, limited the inflammation phase, and improved the antibacterial effect. These preclinical findings suggest that the MT-MAA hydrogel may be a promising conductive dressing for the treatment of chronic wounds by ES. MT-MAA hydrogels may also be promising dressings for treating other diseases (e.g., conductive scaffolds for nerve regeneration [9] and cardiac tissue engineering [58]). The polymerization and conductivity of MTs are influenced by various factors, making them responsive-conductive biomaterials. Clearly, more research is still needed to be carried out about this novel conductive biomaterial.

### Electronic supplementary material

Below is the link to the electronic supplementary material.

Supplementary Material 1

## Data Availability

No datasets were generated or analysed during the current study.

## Declarations

### Author information

#Authors and Affiliations.

Weijing Fan, Xiao Yang, Xiaoming Hu, Renyan Huang, Hongshuo Shi, Guobin Liu.

Department of Vascular Surgery, Shuguang Hospital Affiliated to Shanghai University of Traditional Chinese Medicine, Zhangheng Street, Pu Dong New District, Shanghai 201293, China.
