# Supplementary material for: A novel conductive microtubule hydrogel for electrical stimulation of chronic wounds based on biological electrical wires
Source: J Nanobiotechnology. 2024 May 16;22:258. doi: 10.1186/s12951-024-02524-2 (PMC11097419; doi:10.1186/s12951-024-02524-2)
Supplement: Supplementary file 1 — Supplementary Material 1 [file 12951_2024_2524_MOESM1_ESM.docx]

**A novel** **conductive** **microtubule hydrogel for electrical stimulation of** **chronic wounds based on** **biological electrical wires**

**Weijing Fan** **^a^, Xiao Yang ^a^*****, Xiaoming Hu ^a^, Renyan Huang ^a^, Hongshuo Shi ^a,^*****, Guobin Liu ^a,^***

a. Department of Vascular Surgery, Shuguang Hospital Affiliated to Shanghai University of Traditional Chinese Medicine, Zhangheng Street, Pu Dong New District, Shanghai 201293, China.

*. Corresponding author: Guobin Liu, [15800885533@163.com;](mailto:15800885533@163.com;) Hongshuo Shi, [jf17510413109@163.com;](mailto:jf17510413109@163.com,) Xiao Yang, [yxyx-2010@163.com](mailto:yxyx-2010@163.com;).

**Supporting Information**

**1. Experimental section**

**1.1** **Scanning electron microscopy (SEM) images of the MT-MAA hydrogel and MTs.**

SEM was used to show the structure of the MT-MAA hydrogel. Samples (MT-MAA hydrogel and MT-MAA hydrogel seeded with NIH3T3 cells) for SEM were prepared and conducted according to a previous report[1]. Briefly, the samples were soaked in PBS to expose the inner surface with swelling, cut into small pieces, and then the water was removed with freeze-drying (FD-1A-50, BICOOL, Beijing, China). The samples were coated with gold using gold sputtering (RTSP800, Shanghai, China). The structural morphology of the samples was demonstrated using SEM (JEOL 5900, Beijing, China).

SEM was conducted to show the morphology of MTs as previously reported[2]. The MT (5 µl, 1 mg/ml) solution was applied to glass slides, which were dried naturally overnight. Dried samples were placed on slides using double-sided tape. The dried samples were spired with gold to give them a conductive coating. The surface of MTs was shown using SEM (JEOL 5900, Beijing, China). The SEM analysis was completed by the Biomedical Analysis Center of the Shanghai University of Chinese Medicine.

**1.2 Transmission Electron Microscopy (TEM) of MTs.**

The samples of MTs were prepared for TEM as described in a previous study[3]. Briefly, the MT solution (5 µl, 1 mg/ml) was applied to copper grids (200 mesh, SPI, West Chester, USA) and allowed to adhere for 30 s at 30 °C and 95% relative humidity. The copper grids were blotted for 4 s and plunged into ethane slush. The datasets of MTs were collected and analyzed with SerialEM on a 300 keV TEM (CM-10, Philips Netherlands). The TEM analysis was completed by the Biomedical Analysis Center of the Shanghai University of Chinese Medicine.

**1.3 The** **electrical conductivity of MTs was measured using** **the** **electroorientation method** **in different** **cation conditions.**

The conductivity of MTs was measured under various conditions (different cation conditions and pH) as previously described (each n=8, repeated three times)[4]. The electroorientation chamber was prepared with dimensions of 4 mm×4 mm×0.6 mm using glass. For the orientation experiment, MT solutions (5 ml, 1 mg/ml) with different cation concentrations (CaCl_2_, NaCl, AlCl_3_ (0.01 mM, 0.1 mM, 1 mM, 10 mM)) and pH values (5.5, 6, 6.5, 7.0, 7.5, 8, 8.5) were added to the orientation chamber, and a coverslip was used to complete the ceiling. The motion of MTs was shown under a dark-field microscope (Leica, Germany) with an oil-immersion objective lens. The sinusoidal voltage was generated by a function generator (WAVE Factory 1952, NF Co., Yokohama, Japan), and the AC field (25 V rms 5 MHz) was applied to the MT solution. The electrical conductivity measurements and calculations for MTs under various conditions were reproduced as previously described[4].

**1.4 Depolymerization and polymerization of MTs by fluorescence microscopy under different conditions.**

The MTs (HiLyte 488 dye-conjugated tubulin) were synthesized as described above (Materials and methods section: 2.1.1). The MTs were prepared under different conditions (temperature: 4 °C-50 °C; pH: 1-10; CaCl_2_ (0-50 mM)). The MT solution (500 µl, 1 mg/ml) was applied to the surface of slides and then covered by coverslips. Images of the samples were captured with a fluorescence microscope by an oil-immersion objective lens (Leica, Germany) at different time points in a blinded manner (each n=8) and analyzed using ImageJ 6.0 (repeated three times).

**1.5 MTT assay of NIH3T3 cells in the MT-MAA hydrogel/ES group.**

Briefly, 96-well plates were coated with the MT-MAA hydrogel and MAA-hydrogel (50 ul). NIH3T3 cells (1×10^3^ cells/well, 100 ul) were cultured on the MT-MAA hydrogel/ES group (ES: 30 min/d), MT-MAA hydrogel group, MAA-hydrogel/ES group (ES: 30 min/d), and control group (each n=10). ES was carried out at 10 Hz with a 1 ms pulse width and 200 mV. The proliferation of NIH3T3 cells on the hydrogel was evaluated by the MTT Cell Proliferation and Cytotoxicity Assay Kit (Solarbio Science & Technology Co., Ltd. Beijing, China). The absorbance was measured at different time points using a 36.5 °C plate reader (each n=8, repeated three times).

**1.6 Alizarin Red S staining.**

Briefly, cultured MSCs were washed three times with DPBS and fixed in a solution of 4% paraformaldehyde (PFA) at 4 °C for 15 min (each n=10). Alizarin Red S (Sigma, USA) solution (3% in DPBS) was added. The Alizarin Red S solution was incubated for 15 min at 25 °C. Then, it was rinsed five times with DPBS. Finally, images were captured with a fluorescence microscope (TE2000-E, Nikon).

**1.7 Proliferation of** **MSCs by BrdU experiment.**

Six-well plates were coated with the MT-MAA hydrogel and MAA hydrogel. MSCs (1×105 cells/well) were cultured in the MT-MAA hydrogel/ES group (ES: 30 min), MT-MAA hydrogel group, MAA-hydrogel/ES group (ES: 30 min), and control group (each n=10). ES was carried out with the parameters described above. After 12 h, the cells were washed three times in PBS for 5 min. 1×BrdU labeling medium was added to incubate MSCs for 1 h according to the instructions of the BrdU cell proliferation assay kit (Roche, Shanghai, China). The cells were washed three times in PBS for 5 min and incubated with BrdU antibody (1:200, 2 ml, sc-32323, Santa Cruz Corp., USA) overnight at 4 °C. The cells were washed three times in PBS for 5 min, and secondary antibody (1:800, 2 ml,Alexa Fluor™ 594, Invitrogen Corp., Shanghai, China) was added for 1 h incubation at room temperature. After washing with PBS, nuclei were stained using DAPI. The images were captured with a fluorescence microscope in a blinded manner and analyzed using ImageJ 6.0.

**1.8 Masson staining.**

The wound tissues were harvested and prepared on slides at 1 d, 3 d, 5 d, and 7 d. Slides were washed three times in PBS for 5 min (each n=10). Masson staining (abs9347, absin Corp., Shanghai, China) was conducted to show collagen deposition during wound healing according to the instructions. The images were taken by fluorescence microscopy in a blinded manner and analyzed using ImageJ 6.0.

**1.9 Western blotting**

Western blotting was conducted to analyze the deposition of VEGF, EGF and TGF-β within the wound area at 7 d. Proteins (VEGF, EGF and TGF-β) were obtained from frozen wound tissues with lysis buffer (each n=8). The protein concentration was measured by a BCA protein assay kit (Beyotime, Shanghai, China). The proteins were separated by SDS‒PAGE and transferred to PVDF membranes (Millipore, MA, USA). After transferring, the PVDF membranes were blocked in TBS-T to prevent nonspecific antibody binding and incubated with the primary antibodies for 24 h in 3% milk at 4 °C: (1) polyclonal anti-mouse VEGF (#2463, Cell Signaling Technology, Boston, USA), EGF (#6937, Cell Signaling Technology, Boston, USA) and TGF-β (A2547; Sigma‒Aldrich, St. Louis, MO, USA) and (2) monoclonal anti-mouse GAPDH (Santa Cruz, Dallas, TX) at a 1:1000 dilution as a loading control. The band densities were calculated with Image Laboratory software (Bio-Rad, Hercules, CA, USA).

**2. Results.**

**
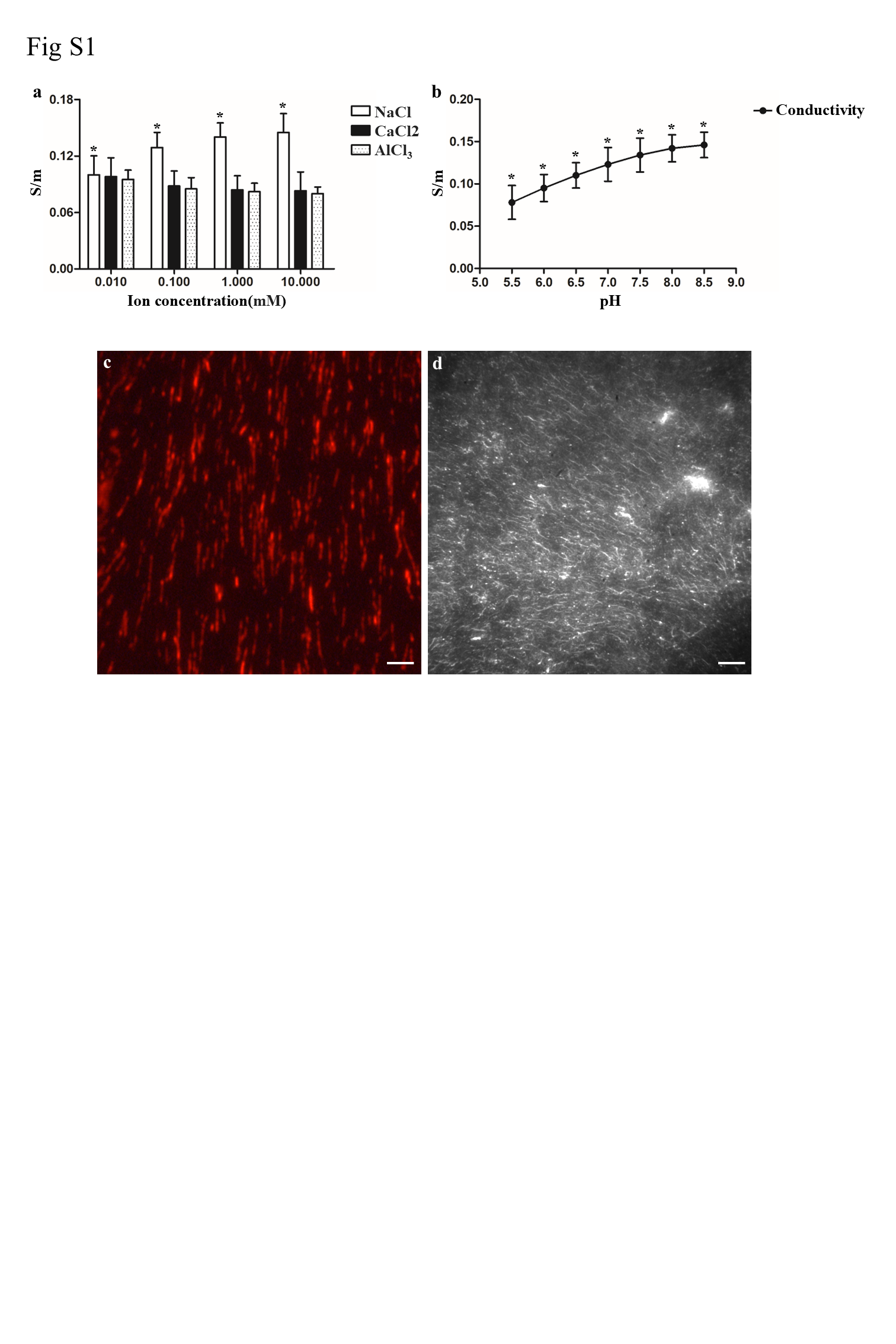
**

**Fig. S1 The conductivity of MTs in** **different** **environments, and MTs were aligned by an electric field in vitro.** (a) Conductivity of MTs under different cation conditions. (b) Conductivity of MTs in different acidic environments. (c, d) Microscopy images of MTs (HiLyte 488 dye labeled) in E-fields. Scale bars=1 μm for (b, c). *p < 0.05, compared among all the groups.


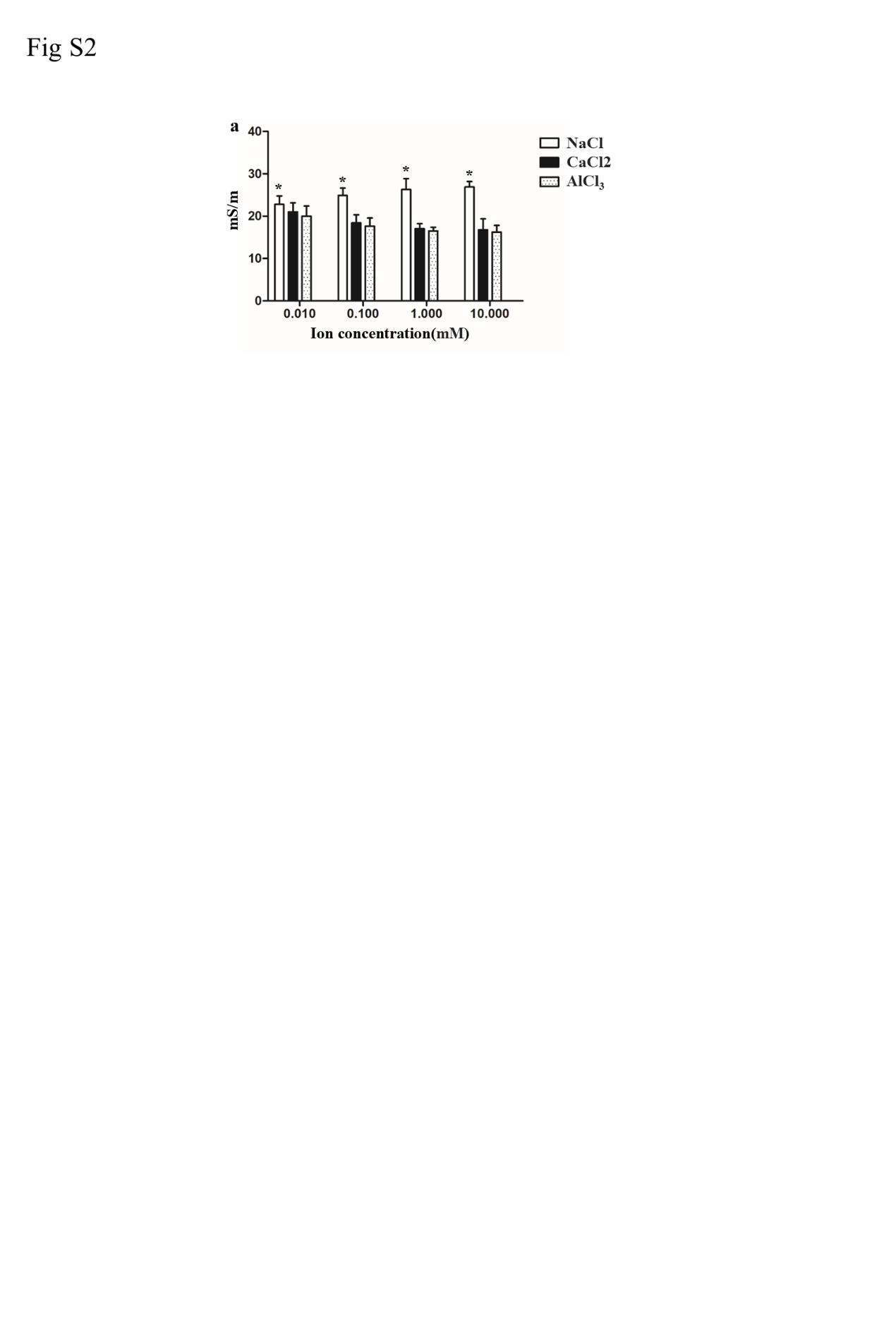


**Fig. S2** **Conductivity of the MT-MAA hydrogel under different** **cation conditions.** (a) Conductivity of the MT-MAA hydrogel under different cation conditions (NaCl, CaCl_2_, and AlCl_3_) at different concentrations. *p < 0.05, compared among all the groups.


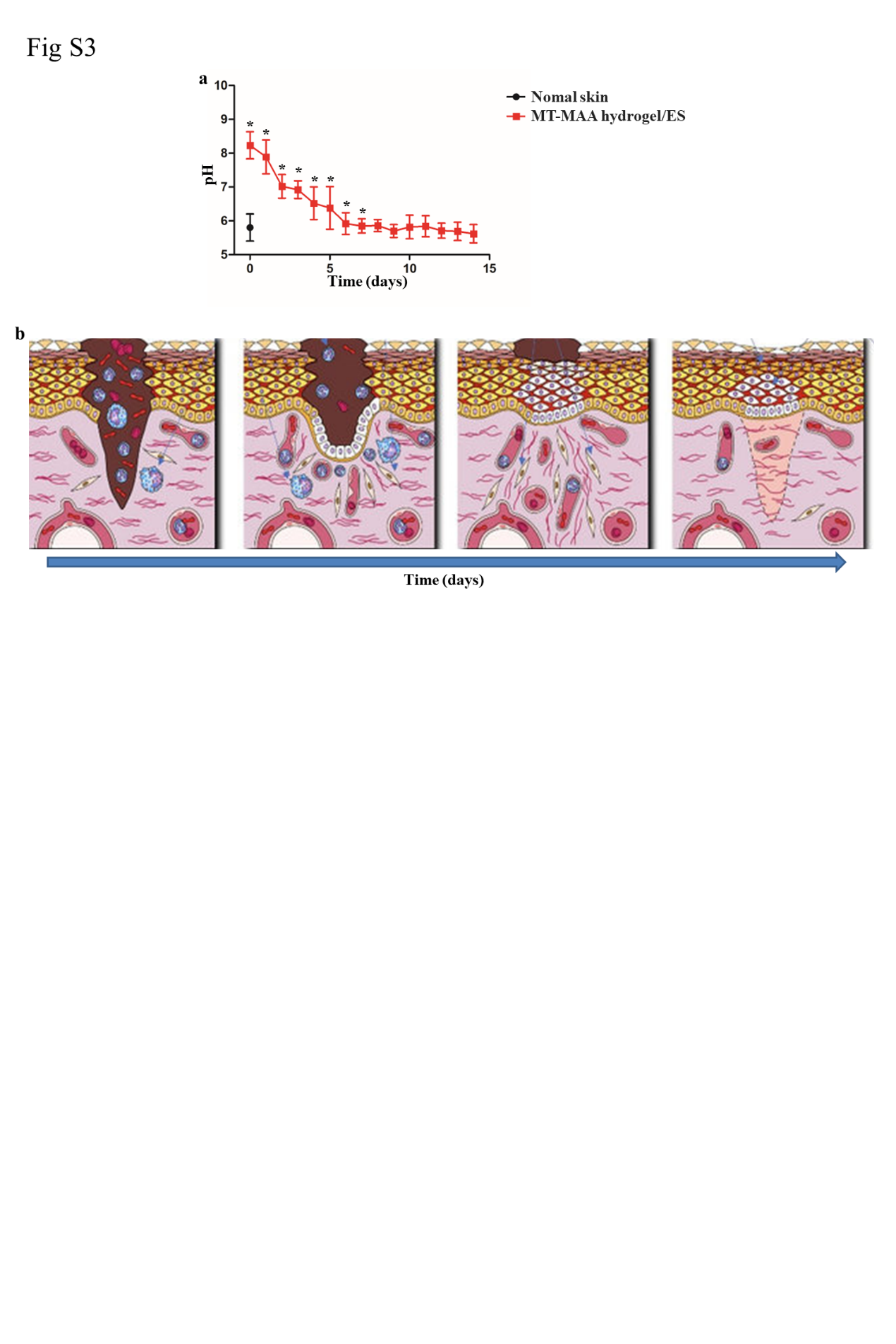


**Fig. S3 The pH of the wound bed in the** **wound healing process.** (a) The pH of the wound bed and normal skin. (b) Scheme of the wound healing process. *p < 0.05, compared among all the groups.

**
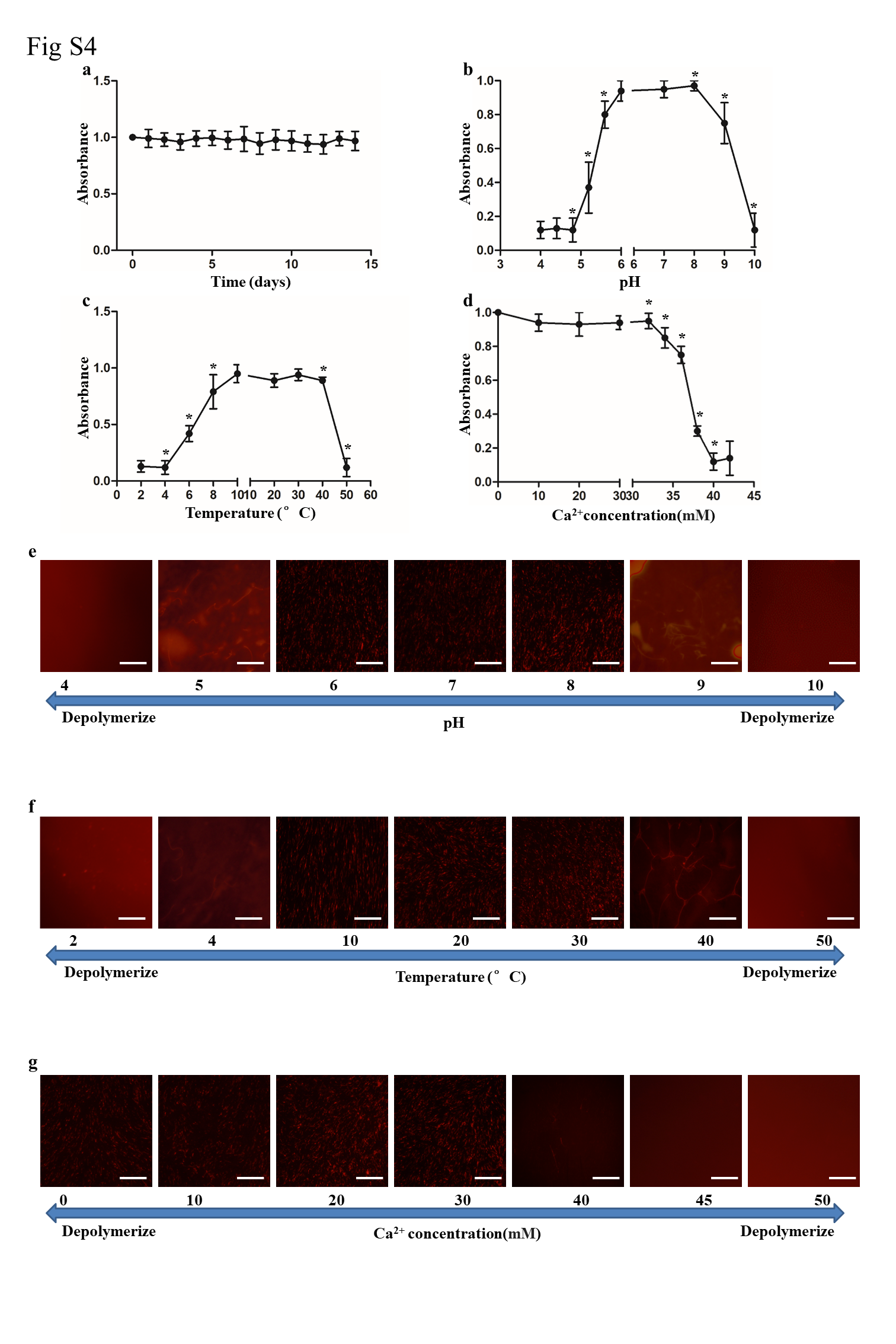
**

**Fig. S4 The effects of different environments on the polymerization of MTs in vitro.** (a) The station of MTs within 14 d in vitro. (b-d) The station of polymerization under different ambient environments by absorbance. (e-g) The station of polymerization under different environments using immunofluorescence. (scale bar=20 μm). *p < 0.05, compared among all the groups.


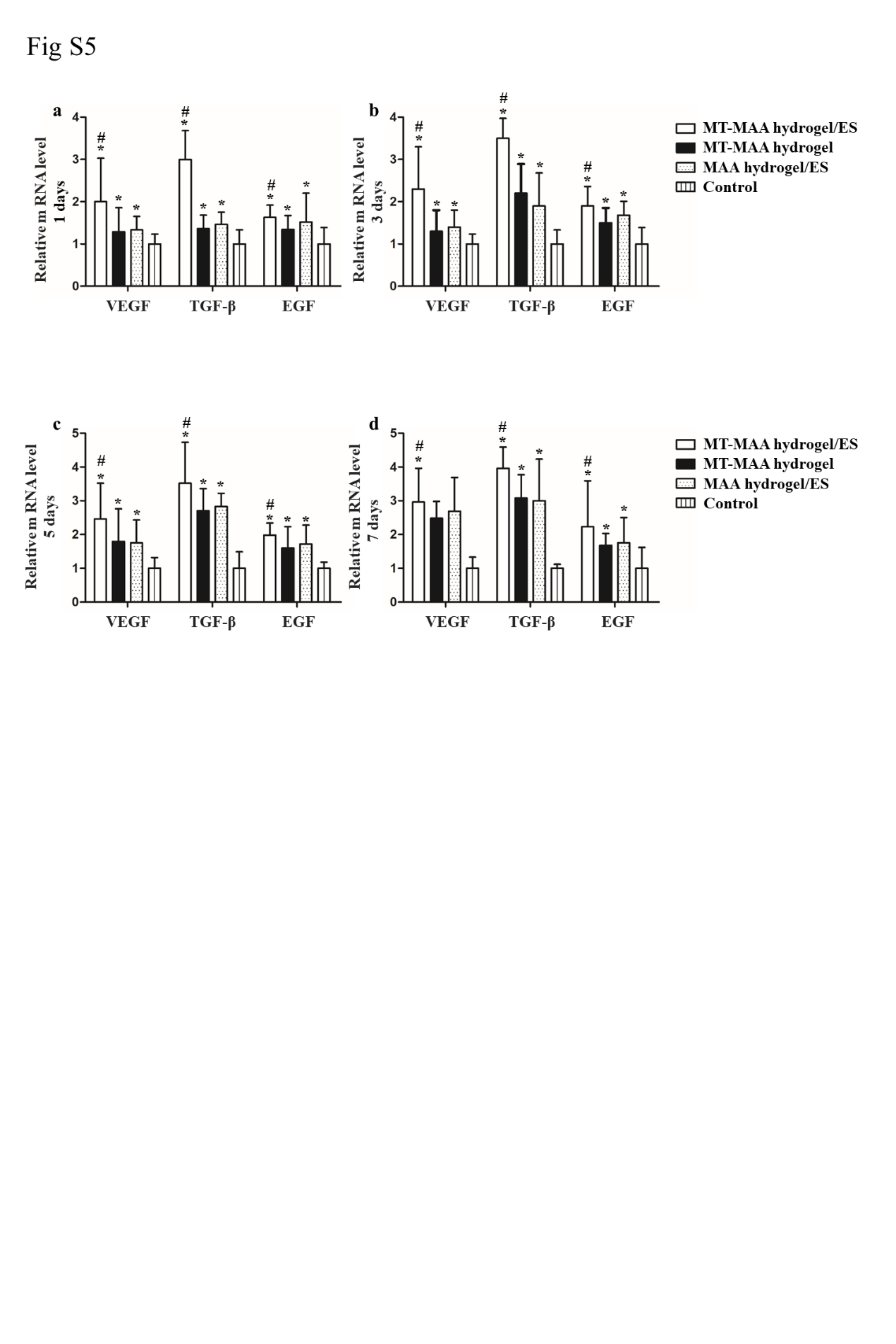


**Fig. S5 The gene expression of growth factors in NIH 3T3 cells i****n different groups.** (a-d) The gene expression of EGF, TGF-β, and EGF at different time points in different groups by RT‒PCR. *p < 0.05, compared with the control group. ^#^p < 0.05, compared with the MT-MAA hydrogel group and MAA hydrogel/ES group.

**
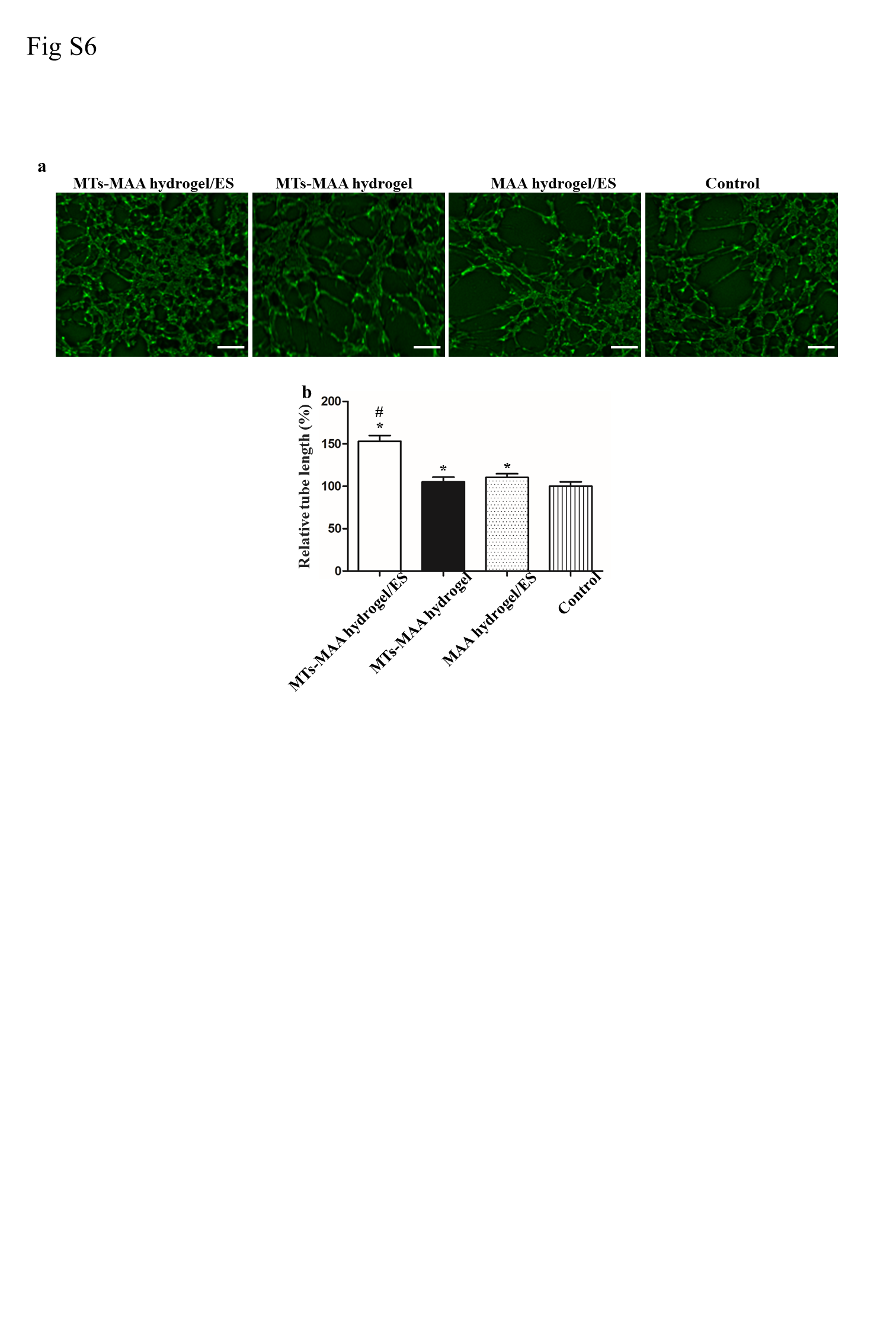
**

**Fig. S6 Tube formation of ECs in different groups.** (a) Fluorescence microscopy images of tube formation in different groups at 3 d. (scale bar=20 μm). (b) Quantification of tube length in different groups. *p < 0.05, compared with the control group. ^#^p < 0.05, compared with the MT-MAA hydrogel group and MAA hydrogel/ES group.

**
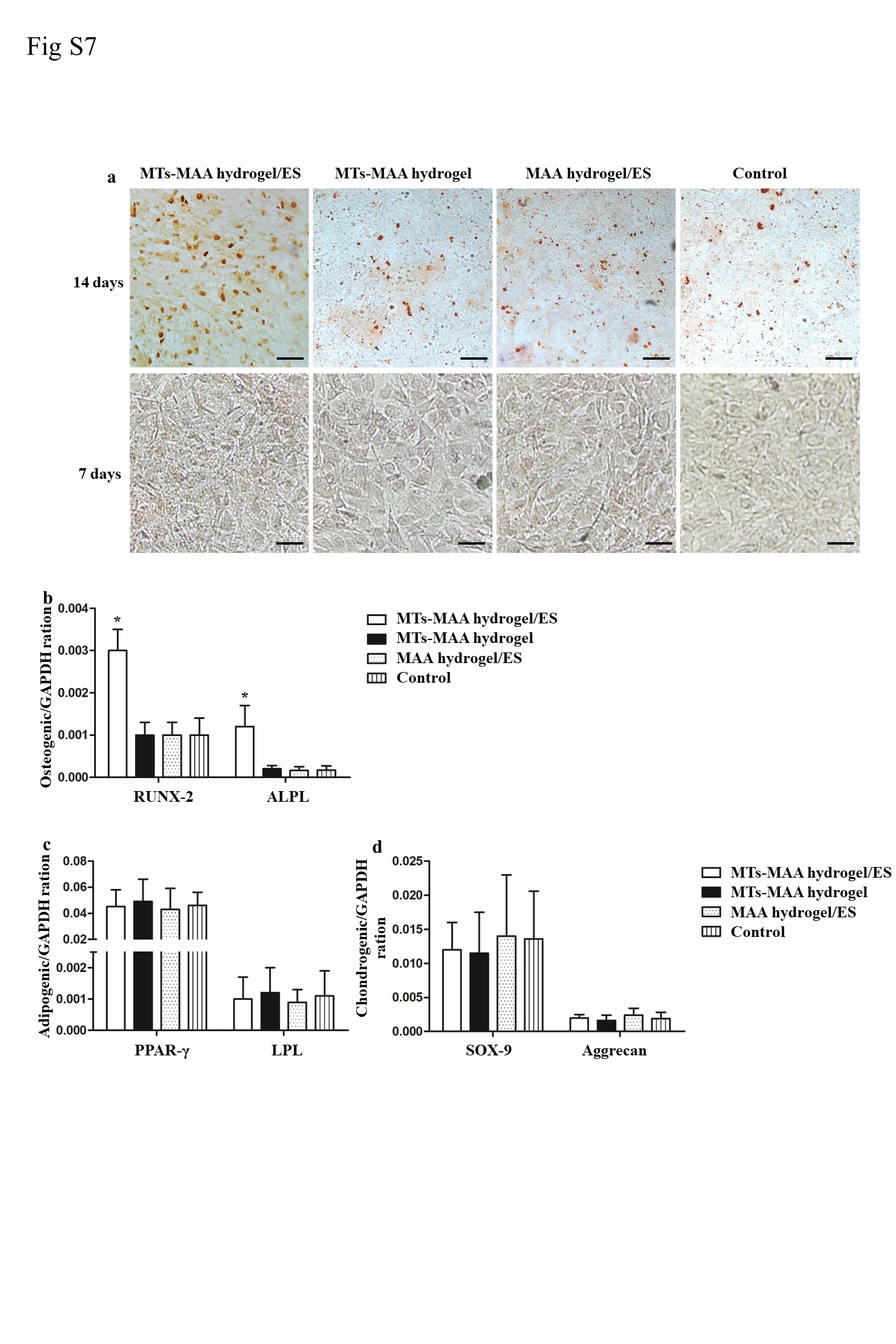
**

**Fig. S7 Osteogenic differentiation of MSCs.** (a) Red S staining of MSCs in different groups. (scale bar=20 μm). (b-d) Gene expression related to the osteogenic, adipogenic, and chondrogenic differentiation potentials of MSCs using RT‒PCR. *p < 0.05.

**
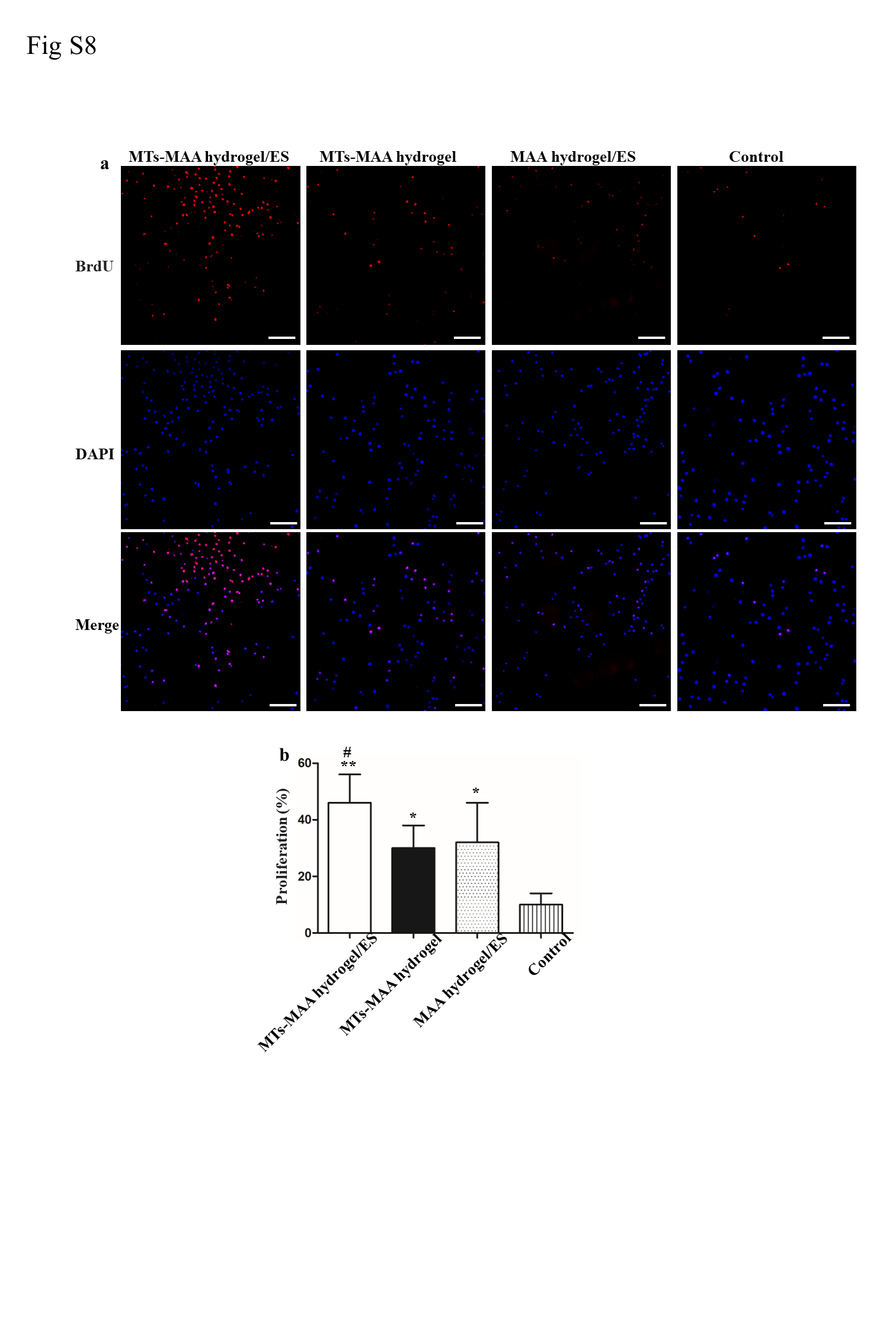
**

**Fig. S8 The effects on the proliferation of MSCs in different groups.** (a) Microscopic images of BrdU experiments, (red) BrdU; (blue) DAPI. Scale bar=50 μm. (b) Quantification of proliferation (%). **p < 0.01, *p < 0.05, compared with the control group. ^#^p < 0.05, compared with the MT-MAA hydrogel group and MAA hydrogel/ES group.

**
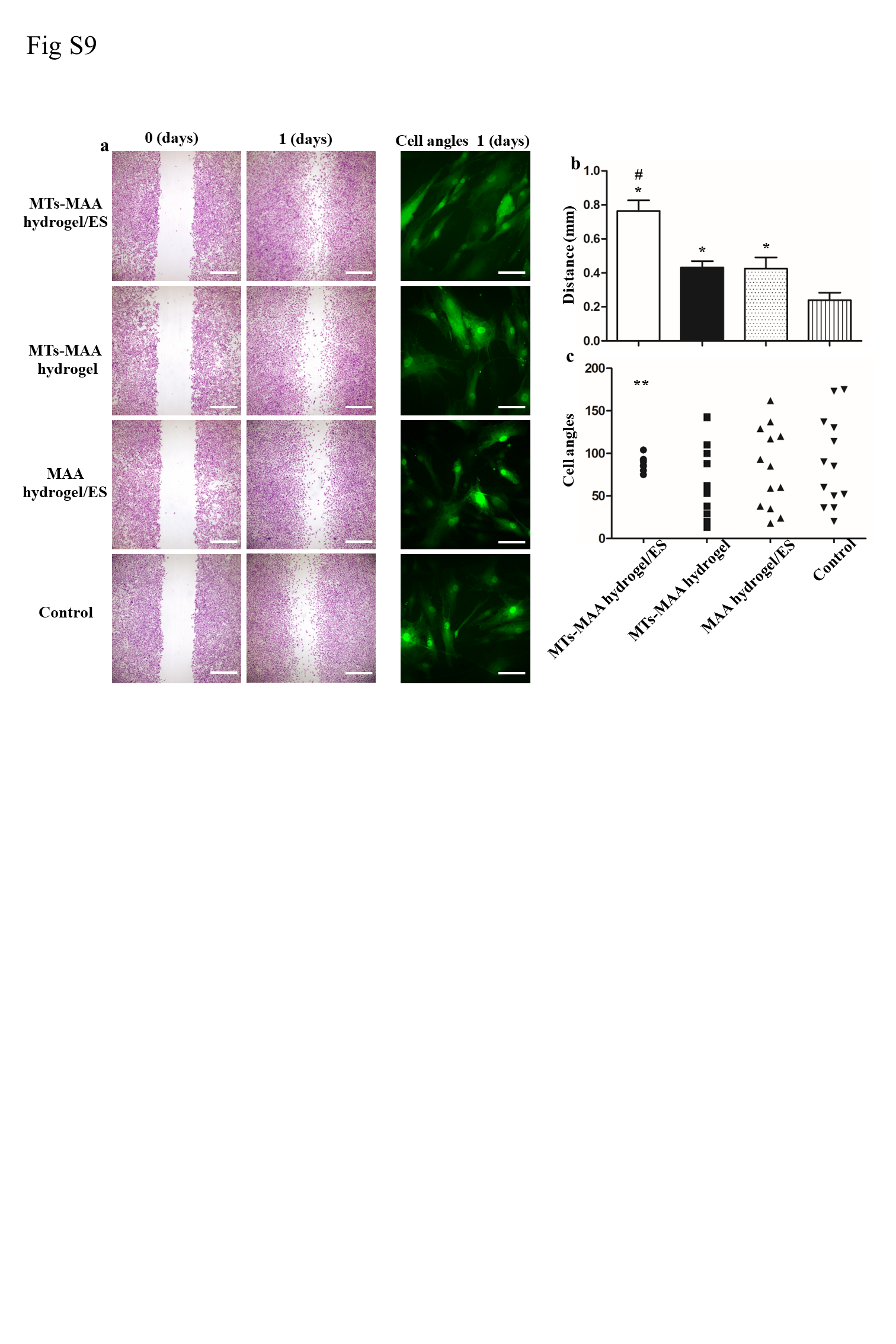
**

**Fig. S9 Enhancement of aligned migration of MSCs in MT-MAA hydrogel applied by ES in vitro.** (a, b) Microscopic images of the scratch assay. (c, d) Quantification of migration distance and angles at 1 d. Scale bars represent 20 μm for (a) and 5 μm for (b). *p < 0.05, **p < 0.01, compared with the control group. ^#^p < 0.05, compared with the MT-MAA hydrogel group and MAA hydrogel/ES group.


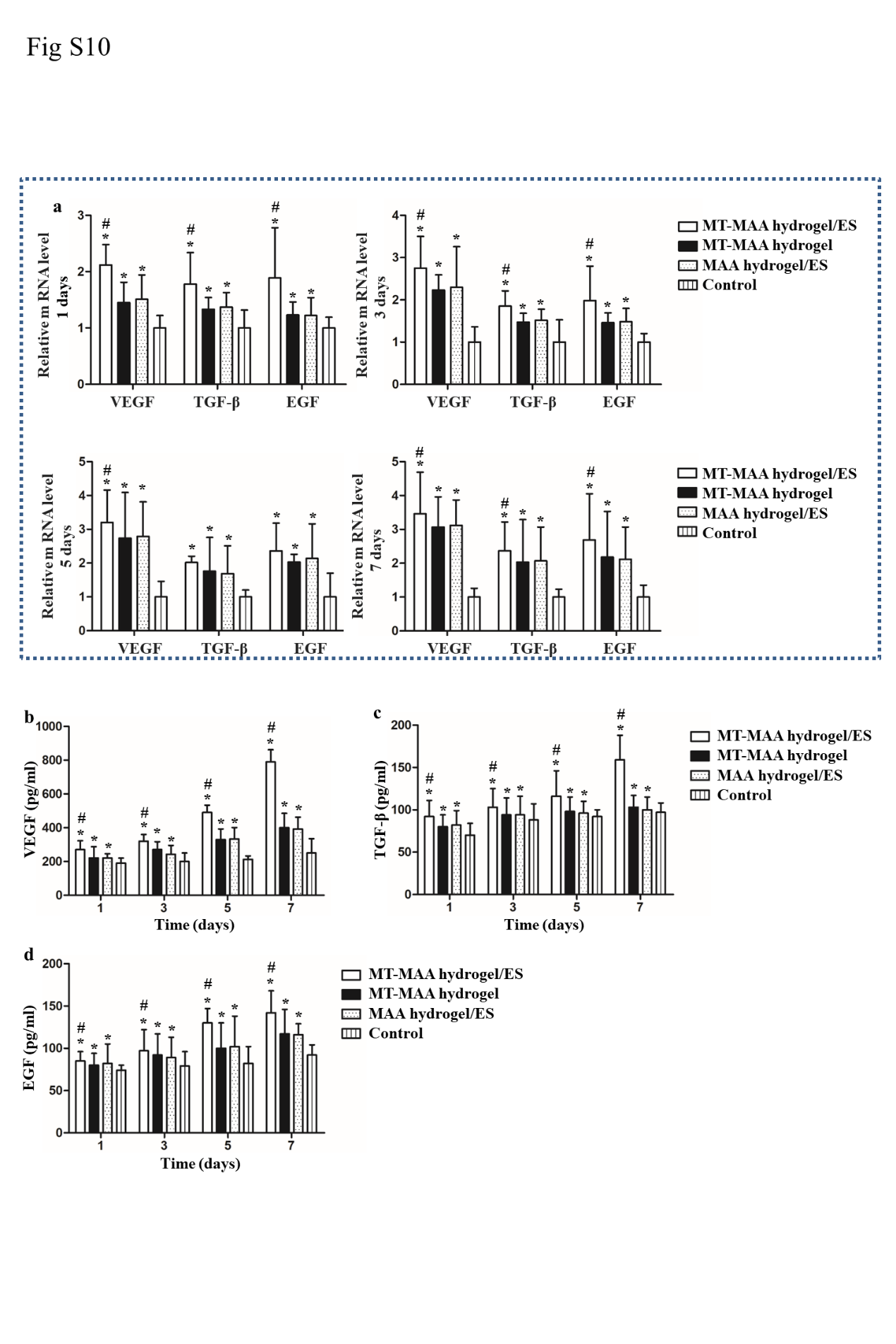


**Fig. S10 The effects of the MT-MAA hydrogel/ES on the** **paracrine effects of MSCs.** (a) The gene expression of growth factors in different groups. (b-d) The secretion of growth factors at different time points. *p < 0.05, compared with the control group. ^#^p < 0.05, compared with the MT-MAA hydrogel group and MAA hydrogel/ES group.

**
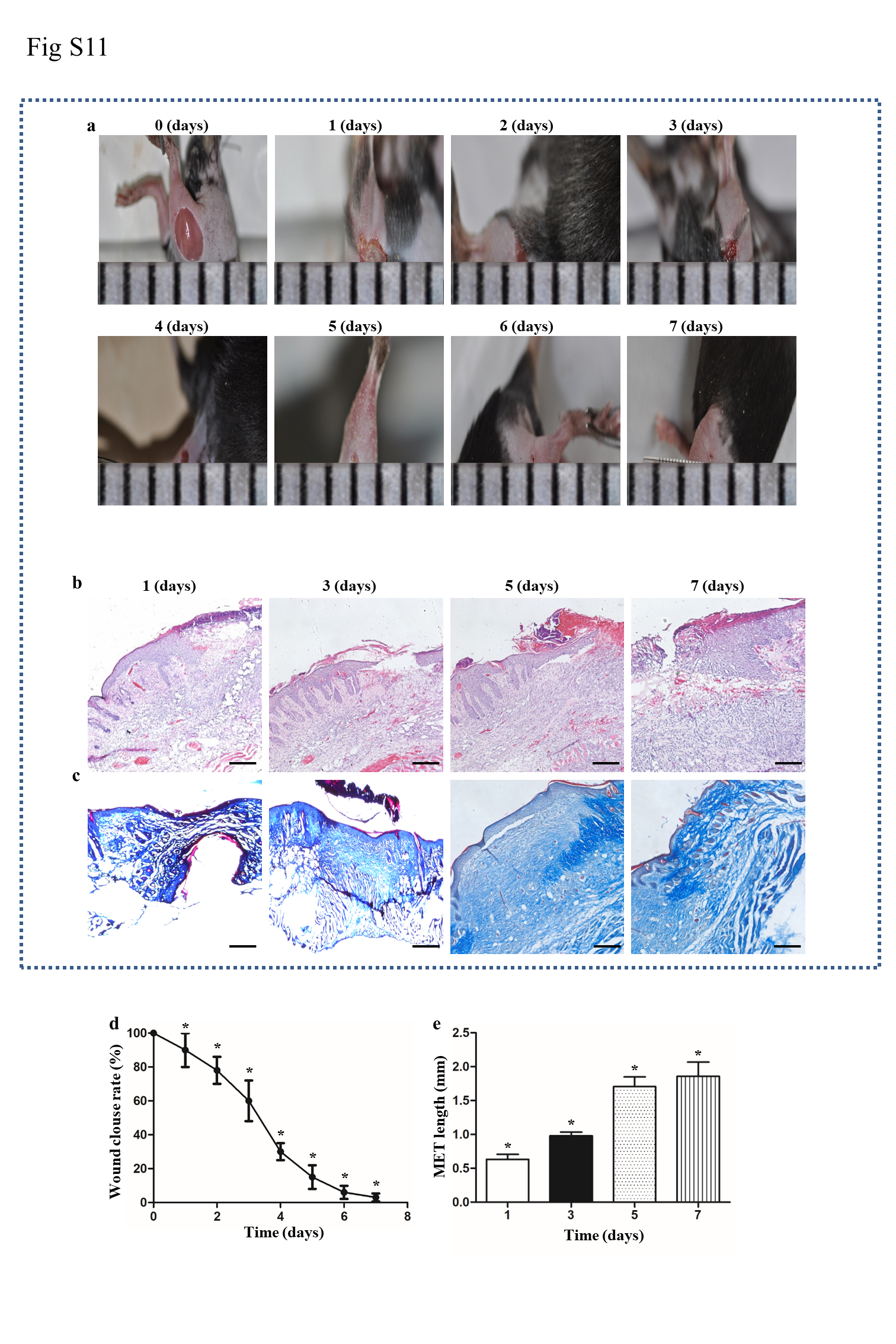
**

**Fig. S11 MSCs seeded in MT-MAA hydrogel/ES revealed rapid wound closure and re-epithelialization.** (a) Images of wounds at 0-7 d. (b) H&E staining of granulation tissue at 1 d, 3 d, 5 d, and 7 d (scale bar = 1 mm). (c) Masson staining of wounds at 1 d, 3 d, 5 d, and 7 d (scale bar = 1 mm). (d, e) Quantification of wound closure and MET length. *p < 0.05, compared among all the groups.

**
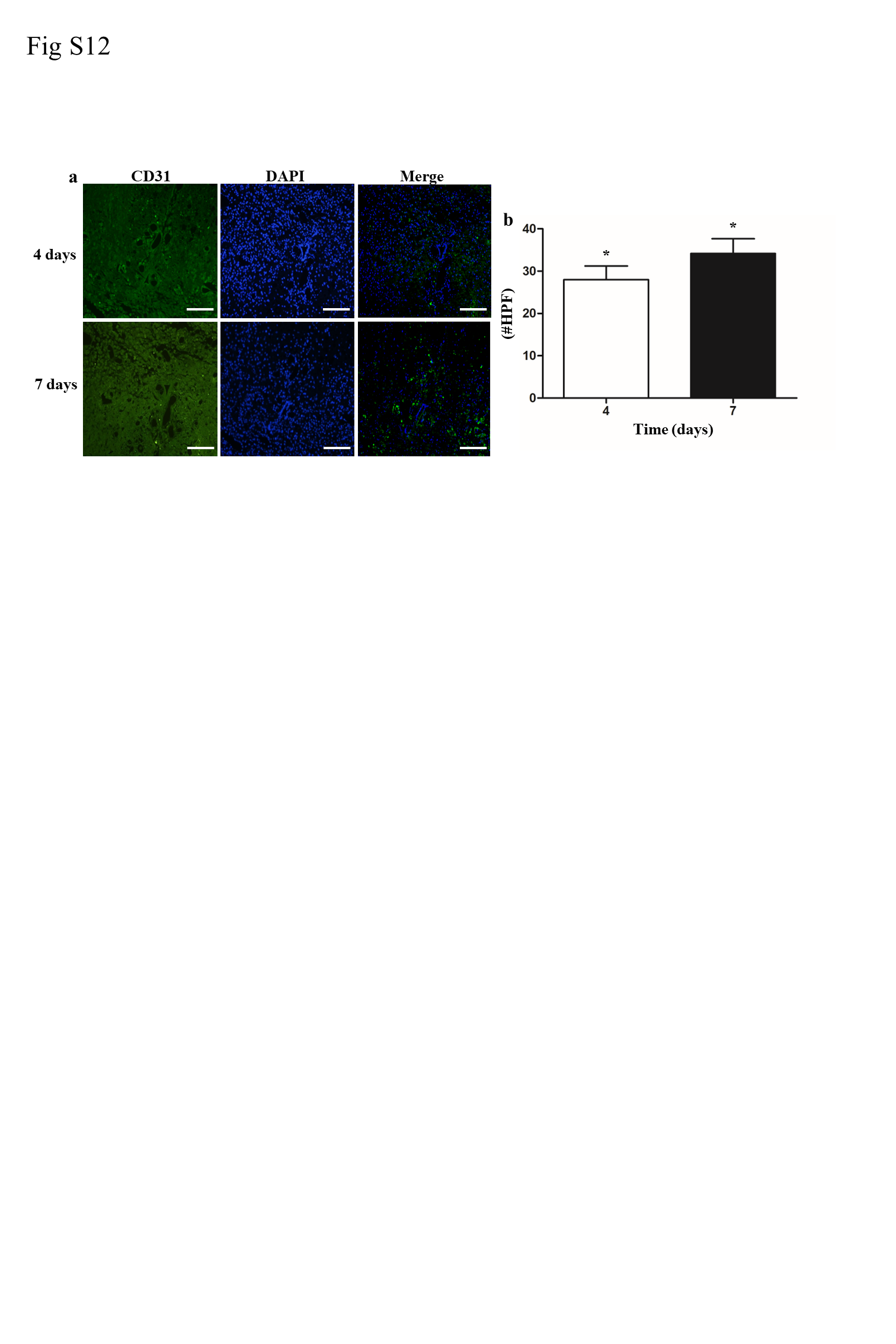
**

**Fig. S12 Improvement of** **angiogenesis in MT-MAA hydrogel/ES seeded with MSCs.** (a) Staining of CD31-positive microvessels of wounds at 4 and 7 d, (green) CD31; (blue) DAPI; (scale bar=100 μm). (b) Quantification of angiogenesis. *p < 0.05.

**
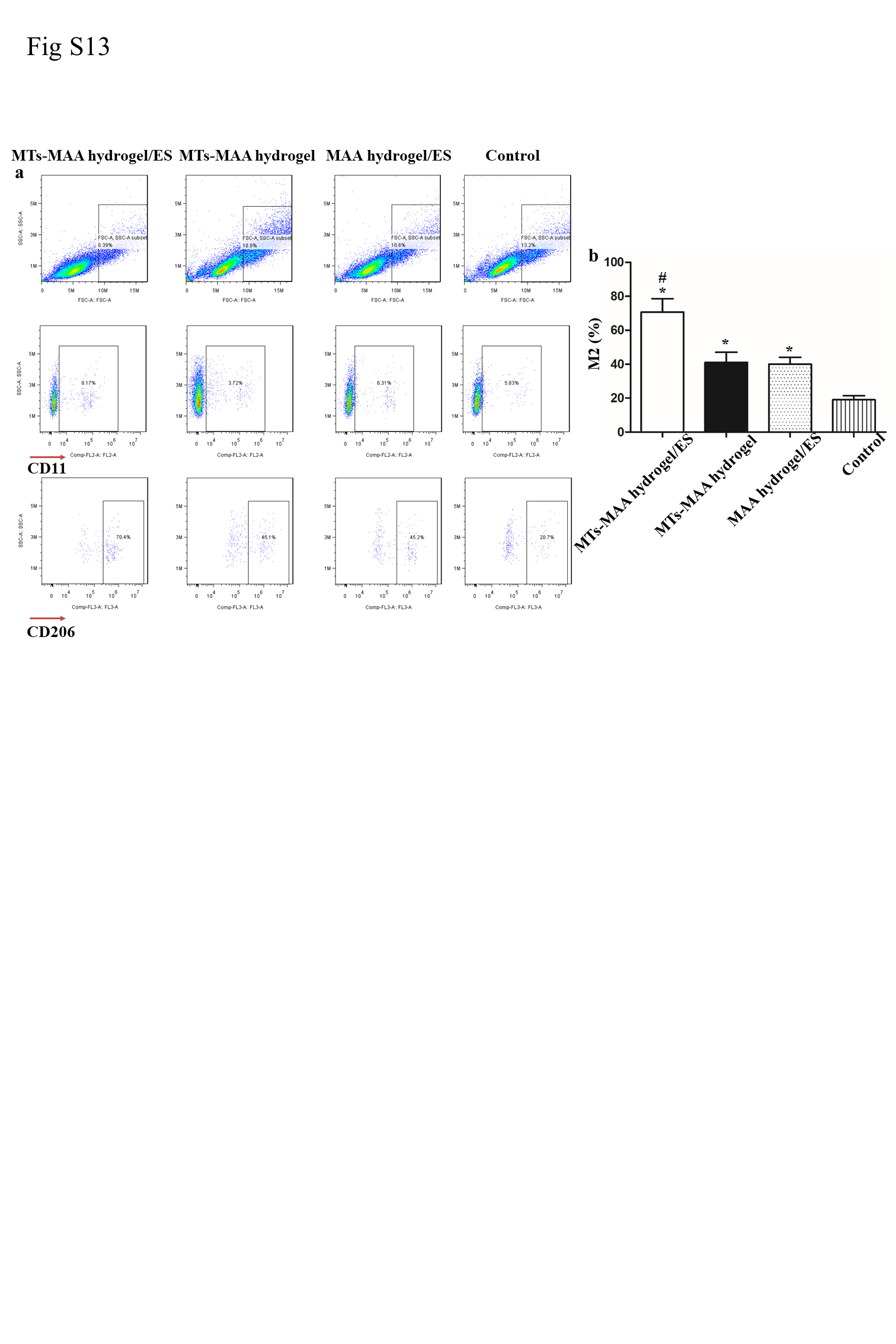
**

**Fig. S13** **Macrophage phenotype from pro-inflammatory/M1 toward a pro- repair/M2 performed by flow cytometry.** (a) Macrophage phenotype at 4 d by flow cytometry. (b) Quantification of the M2 phenotype in different groups. *p < 0.05, compared with the control group. ^#^p < 0.05, compared with the MT-MAA hydrogel group and MAA hydrogel/ES group.


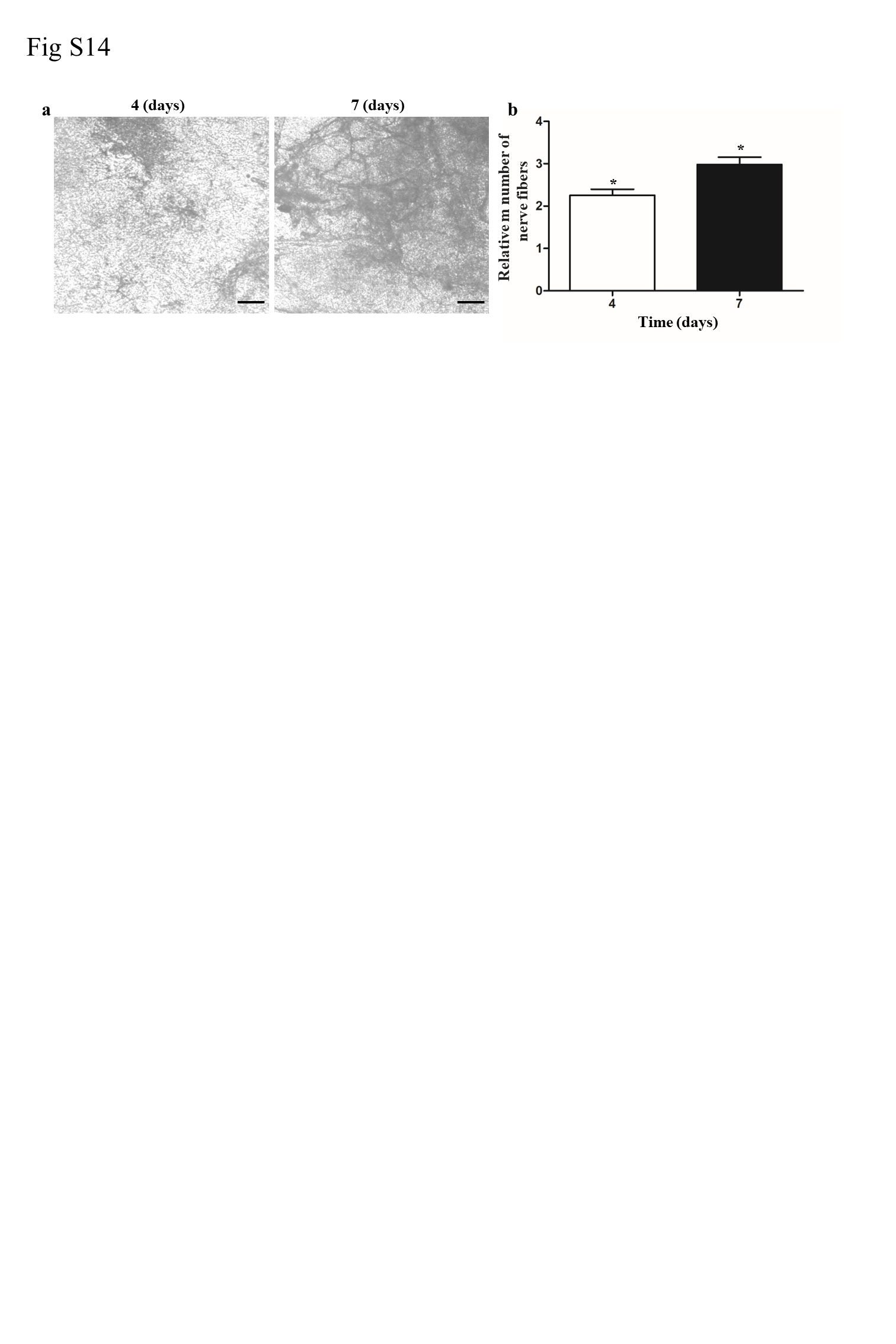


**Fig. S14 Acceleration of nerve growth in MT-MAA hydrogel/ES seeded with MSCs.** (a) Images of silver staining at 4 and 7 d (scale bar=50 μm). (b) Quantification of nerve fiber number. *p < 0.05.

**Table S1 the primer of VEGF, TGF-𝛽, EGF and related genes of MSCs differentiation.**

| **Gene** | **Forward prim(5^,^-3^,^)** | **Reverse primer(5^,^-3^,^)** |
| --- | --- | --- |
| RUNX2 | GTGCCTAGGCGCATTTCA | CACCTGCCTGGCTCTTCTTA |
| ALPL | GGGTCAGCTCCACCACAA | GCATTGGTGTTGTACGTCTTG |
| PPAR-γ | GGTGAAACTCTGGGAGATCC | TGAGGGAGTTTGAAGACTCTTC |
| LPL | TCAACTGGATGGAGGAGGAG | GGGGCTTCTGCATACTCAAA |
| SOX9 | GAGCAGACGCACATCTC | CCTGGGATTGCCCCGA |
| Aggrecan | TCGAGGACAGCGAGGCC | TCGAGGGTGTAGCGTGTAGAGA |
| VEGF | TGCATTCACATTTGTTGTGCTGTAG | GCAGATTATGCGGATCAAACC |
| TGF-𝛽 | CGAGTCCTGTAGGATCGCCAT | ATTCAAGTCAACTGTGGAGCAAC |
| EGF | TCAGGCTGGAAGGAGAAGATGC | AACTGTGTCATTCCAGGATC |
| GAPDH | GGTGAAGGTCGGAGTCAACGG | GGTCATGAGTCCTTCCACGAT |

**Table S2 Quasistatic compressive and tensile properties of** **MTs-MAA hydrogel with various MAA content at room temperature (n=4).**

| **Samples**  **(MAA%)** | **Compression** | | | | **Tension** | | |
| --- | --- | --- | --- | --- | --- | --- | --- |
|  | **σ_Com_(MPa)** | **ε_Com_** | **E_Com_(MPa)** |  | **σ_Com_(MPa)** | **ε_Com_** | **E_Com_(MPa)** |
| 0.5 | 1.12±0.08 | 2.41± 0.13 | 4.26± 0.11 | | 0.13± 0.02 | 3.68± 0.12 | 0.68± 0.08 |
| 1 | 1.22± 0.04 | 2.72± 0.08 | 4.53± 0.12 | | 0.19± 0.01 | 4.54±0.15 | 0.69±0.09 |
| 1.5 | 1.34± 0.06 | 3.21± 0.11 | 4.82± 0.09 | | 0.21± 0.03 | 5.16± 0.08 | 0.72± 0.10 |
| 2 | 1.46± 0.12 | 3.48± 0.10 | 5.27±0.13 | | 0.29± 0.03 | 5.52± 0.10 | 0.81± 0.08 |

**References**

[1] J. Su, S.C. Satchell, J.A. Wertheim, R.N. Shah, Poly(ethylene glycol)-crosslinked gelatin hydrogel substrates with conjugated bioactive peptides influence endothelial cell behavior, Biomaterials 201 (2019) 99-112.

[2] I. de Andrade Rosa, W. de Souza, M. Benchimol, High-resolution scanning electron microscopy of the cytoskeleton of Tritrichomonas foetus, J Struct Biol 183(3) (2013) 412-418.

[3] P. Kiesel, G. Alvarez Viar, N. Tsoy, R. Maraspini, P. Gorilak, V. Varga, A. Honigmann, G. Pigino, The molecular structure of mammalian primary cilia revealed by cryo-electron tomography, Nat Struct Mol Biol 27(12) (2020) 1115-1124.

[4] I. Minoura, E. Muto, Dielectric measurement of individual microtubules using the electroorientation method, Biophys J 90(10) (2006) 3739-48.
